# Genomic insights into biosynthesis and adaptation in the bioactive marine bacterium *Streptomyces albidoflavus* VIP-1 from the Red Sea

**DOI:** 10.1186/s12866-025-04109-x

**Published:** 2025-06-26

**Authors:** Abdelrahman M. Sedeek, Hossam Elfeky, Amro S. Hanora, Samar M. Solyman

**Affiliations:** 1 Department of Microbiology and Immunology, Faculty of Pharmacy, Galala University, New Galala City, Suez 43511 Egypt; 2 Department of Microbiology and Immunology, Faculty of Pharmacy, Suez Canal University, Ismailia, Egypt; 3 Department of Microbiology and Immunology, Faculty of Pharmacy, King Salman International University-Ras Sudr, Ras Sudr, Egypt; 4 Department of Microbiology and Immunology, Faculty of Pharmacy, Sinai University- Elkantara branches, Ismailia, Egypt

**Keywords:** Marine actinobacteria, Antimicrobial, Anticancer, Symbiotic bacteria, Microbial genomics

## Abstract

**Background:**

Marine actinobacteria represent a diverse and biotechnologically rich group of microorganisms that have adapted to the unique challenges of marine ecosystems, including fluctuating salinities, temperatures, pressures, and nutrient levels. These environmental pressures have enhanced their biosynthetic capabilities, making them a prolific source of novel bioactive compounds.

**Results:**

In this research, we report the isolation of a novel marine bacterium “*Streptomyces albidoflavus* VIP-1” associated with the marine invertebrate *Molgula citrine* isolated from the Red Sea. The secondary metabolites from the isolated strain exhibited significant in vitro antimicrobial and antitumor activities. The isolate has an estimated genome length of 7,090,100 base pairs. Based on the phylogenomic analysis and the values of digital DNA-DNA hybridization, average amino acids identity, and average nucleotide identity in comparison to genomes of known type strains, the isolated strain was found to belong to the species of *Streptomyces albidoflavus*. The genome of *S. albidoflavus* VIP-1 revealed genetic adaptations enabling its survival in harsh environments, including stress response genes and regulatory systems. Moreover, a wide variety of biosynthetic gene clusters belonging to polyketides, terpenes, and non-ribosomal peptides were detected. Finally, a comparative genome analysis with related marine and terrestrial strains highlighted its elevated biosynthetic potential.

**Conclusions:**

The genome of *S. albidoflavus* VIP-1 reflects its potential as a valuable resource for biotechnological and biomedical applications. It reveals genetic adaptation to the marine environment through various anti-stress mechanisms and competitive strategies, including the production of antimicrobial metabolites.

**Supplementary Information:**

The online version contains supplementary material available at 10.1186/s12866-025-04109-x.

## Introduction

Marine microorganisms have long been recognized as a rich source of novel bioactive molecules, serving as a cornerstone for many pharmaceutical and biotechnological applications. These microbial communities have a remarkable ability to synthesize a huge diversity of complex chemical compounds, many of which possess potent biological activities. From antibiotics and antitumors to immunosuppressants and anti-inflammatory drugs, marine microorganisms continue to be a valuable source of novel therapeutic agents [1].

Marine microbial communities are exposed to harsh conditions such as lack of light, high salinity, nutrient limitation, extreme pH and pressure, highly variable weather conditions, and predator attacks, which work as a driving force not only for the production of microbial bioactive secondary metabolites but also the evolution of diverse metabolic pathways [2].

Marine tunicates are known to harbor diverse microbial communities within their tissues, forming complex symbiotic relationships. These symbionts can contribute to host defense, nutrient exchange, and chemical signaling. Several studies have reported that tunicate-associated bacteria are prolific producers of secondary metabolites, some of which may be involved in deterring predators or competing pathogenic microorganisms [3]. The relationship between tunicates and their associated microbes creates a specialized environment that encourages the development of new biosynthetic pathways in the microbes [4].

The Red Sea is considered a unique marine ecosystem for several reasons: high temperature, high salinity, high evaporation rate, and low river inflow [5]. It also has a unique, diverse, and rich coral reef system [6]. Marine invertebrates such as sponges, tunicates, soft and hard corals, bryozoans, sea slugs, and others account for more than 89% of all extant organisms in the marine environment, represented by over 174,600 species [7]. The diversity of symbiotic microorganisms associated with Red Sea marine invertebrates and their ability to produce pharmaceutically and biotechnologically promising bioactive compounds have been reported in previous studies from our laboratory [8–11].

There are several successful examples of discovering novel bioactive metabolites with promising applications from host-associated microbes from the Red Sea. For instance, Kamel et al. (2021) screened crude extracts of Red Sea marine sponges and their associated bacterial extracts for antimicrobial activity. While crude sponge extracts showed activity in four out of five species, a remarkable 84 out of 110 bacterial isolates derived from these sponges exhibited potent antimicrobial effects [10]. In 2023, Elfeky et al. screened Red Sea nudibranch-associated bacteria for antimicrobial and anticancer activity and showed that bacteria associated with Red Sea nudibranchs are highly bioactive and have antimicrobial and antitumor activities [11].

In bacterial genomes, biosynthetic gene clusters (BGCs) encode the enzymatic machinery responsible for the production of secondary metabolites. Among these, polyketide synthases (PKSs) and non-ribosomal peptide synthetases (NRPSs) are two major classes, known for synthesizing structurally complex and bioactive compounds [9, 12]. These BGCs often consist of modular, multi-domain enzymes that can be rearranged or hybridized, resulting in a vast chemical diversity [13, 14].

Actinobacteria (phylum Actinomycetota) are known as factories of bioactive secondary metabolites. Marine ecosystems represent promising habitats for discovering novel strains of actinobacteria, making them promising resources for novel microbial bioactive metabolites [15]. Among the various genera of marine actinobacteria, *Streptomyces* stands out as a prolific producer of bioactive compounds. This genus is well-known for its ability to synthesize a vast diversity of secondary metabolites with biological activities. For instance, Liu et al. (2024) reviewed over 260 novel alkaloids isolated from marine *Streptomyces* and reported between 2013 and 2023, with approximately 60% exhibiting significant antibacterial, anticancer, or anti-inflammatory activities. These compounds had various structural classes, including indoles, pyrroles, and pyridines, underscoring the chemical diversity inherent to marine *Streptomyces* [16]. Although genome mining in *Streptomyces* reveals numerous BGCs (averaging 44, with some strains containing up to 83 BGCs), the majority remain unexpressed under standard laboratory conditions [17]. Traditional approaches, which include cultivation, bioassay-guided fractionation, and systems-level perturbations (e.g., media changes) to induce metabolite production without prior genome analysis for its metabolic capabilities, often result in the rediscovery of known compounds and are limited by the inability to activate the majority of silent biosynthetic pathways [18]. In contrast, the application of advanced genomic tools has facilitated the identification of these silent BGCs, paving the way for the discovery of novel natural products with potential therapeutic applications [17, 19]. For example, Yang et al. (2020) reviewed the importance of whole-genome sequencing and recent bioinformatics tools in drug discovery from marine microbial natural products. This study highlighted the recent dependency of researchers on the use of bioinformatics to analyze the genome of marine *Streptomyces* species, which has helped them find promising new bioactive molecules, including novel antimicrobial agents. This approach is much faster than traditional methods and accelerates the discovery of new drugs [20].

In addition to their high capacity for the production of secondary metabolites with antimicrobial or signaling functions, marine-derived *Streptomyces* have evolved various ecophysiological adaptations that enable them to survive in marine environments, including the production of osmoprotectants and salt-tolerant enzymes [21]. Genomic analyses have also revealed the presence of genes involved in oxidative stress resistance, metal ion homeostasis, and membrane transport systems, which collectively contribute to their resilience in marine ecosystems [21, 22].

The current study focuses on *Streptomyces albidoflavus* VIP-1, a bioactive marine bacterium isolated from the marine tunicate *Molgula citrina* collected from the Red Sea. This strain was selected for further investigation due to its notable in vitro antimicrobial and antitumor activities observed in preliminary screenings. Given the unique conditions of the Red Sea, microorganisms from this region are likely to possess unique biosynthetic pathways and stress-adaptation mechanisms. Therefore, to explore the genomic basis of its bioactivity and environmental adaptation, we performed a comprehensive whole-genome sequence analysis of this marine bacterium to elucidate its phylogenetic relationships, genome characteristics, and biosynthetic potential, and to compare it with its nearest relatives to gain a better understanding of the biosynthetic potential of marine *Streptomyces* species.

## Materials and methods

### Sample collection and strain isolation

**Fig. 1:**
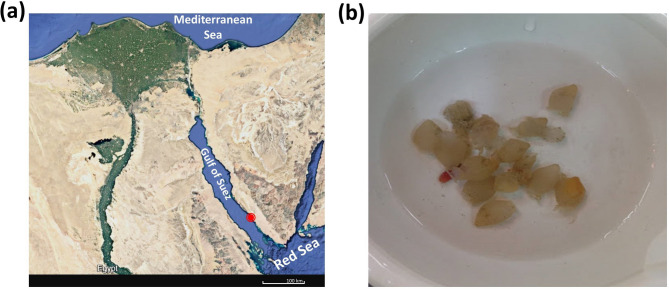
Marine invertebrate *Molgula citrine* sample collection (**a**) A map of Egypt indicates the sample collection site (marked in red). (**b**) A picture shows the collected samples of the marine invertebrate *Molgula citrine*

The strain VIP-1 was isolated from the marine invertebrate, *Molgula citrina* (order Pleurogona, family Ascidiidae) collected from the Red Sea (2–7 m depth) at El-Tor, Sharm El-Sheikh, Sinai, Egypt in 2018 (Fig. 1). Samples were collected in sterile bags, transported to the laboratory within 8 h, and maintained at ambient seawater temperature. Approximately 0.5 g of the invertebrate tissue was aseptically excised, rinsed twice with filtered natural seawater (NSW), and homogenized in 9 mL of NSW. Serial dilutions (10⁻¹ to 10⁻⁶) were prepared in NSW. Aliquots of 100 µL were plated onto Reasoner’s 2A agar (R2A) (Difco™, Detroit, USA) supplemented with 2% NaCl and incubated at 30 °C for 1–4 weeks. Isolated colonies were purified through successive streaking and stored as glycerol stocks containing spore suspensions at − 80 °C.

### Preparation of VIP-1 metabolic extract and screening of antimicrobial and antitumor activities

The isolate was cultivated in 250 mL flasks containing 100 mL of R2A broth medium, and prepared with deionized water (Type 1). After 14 days of incubation at 25 °C on an incubator shaker at 220 rpm, the fermented broths were extracted twice with two volumes of ethyl acetate (200 mL × 2). The solvent extracts were evaporated to dryness under vacuum using a rotary evaporator at 40 °C and 170 mbar.

For antimicrobial activity screening, the extracted residual material was dissolved in 15% dimethylsulfoxide (DMSO) to a final concentration of 1 mg/mL. The standard well-diffusion method was used to investigate the antimicrobial activity. A volume of 100 µL of the crude extract was tested against Gram-negative bacteria (*Escherichia coli* ATCC 10536 and *Pseudomonas aeruginosa* ATCC 25619), Gram-positive bacteria (*Staphylococcus aureus* ATCC 9144), and yeast (*Candida albicans* ATCC 90028). 15% DMSO was used as a negative control. Ceftazidime and imipenem were used as positive controls for Gram-negative bacteria, ampicillin was used as a positive control for *S. aureus*, and nystatin was used as a positive control for *C. albicans*.

For antitumor activity screening, the MTT assay was used against human hepatocellular carcinoma (HepG2) and non-small cell lung adenocarcinoma (A549) cell lines as described in Tantawy et al. (2020) [23] with some modifications. Briefly, cancer cells were inoculated into 96-well plates (1 × 10^4^ cells/well) in triplicate and allowed to adhere for 24 h. A stock solution of the metabolic extract was prepared by dissolving the extracted residual material in DMSO (10 mg/mL). Serial dilutions were performed in a complete medium to obtain final concentrations of 1, 10, 100, and 1000 µg/mL, ensuring that the final DMSO concentration never exceeded 0.2% (v/v). After 24 h, the media was replaced with a fresh medium containing the desired concentration of the tested extract. Cells were incubated for an additional 48 h. Cell viability was assessed using the MTT assay. Four hours before the end of the incubation period, 10 µL of MTT solution (5 mg/mL in PBS w/o Ca, Mg, Lonza Verviers SPRL, Belgium) was added to each well. Following incubation, 100 µL of DMSO was added to dissolve the formazan crystals. Absorbance was measured at 490 nm using a microplate reader. Cell viability was calculated as a percentage of the control using the following formula:

 $$\%\;cell\;viability\;=\;(Mean\;absorbance\;in\;test\;wells\;/\;Mean\;absorbance\;in\;control\;wells)\;\times\;100$$

The effect of the tested extract on the morphology of treated liver and lung cancer cells was investigated under a light microscope (Olympus, Japan).

### VIP-1 genomic DNA extraction and whole-genome sequencing

DNA extraction was performed according to the manufacturer’s recommended protocol for the QIAamp DNA Mini Kit (QIAGEN^®^, Germany) with slight modifications to ensure cell lysis. Briefly, 2 µL of 20 mg/mL lysozyme and 40 µL of 20 mg/mL proteinase K were added to the lysis buffer, following the manufacturer’s instructions. A NanoDrop™ 1000 Spectrophotometer V3.7 (Thermo Fisher Scientific Inc., Wilmington, DE, USA) was used to measure the concentration and purity of the DNA. The genomic DNA was sent into DNA stable tubes for whole-genome shotgun Illumina sequencing (IGA Technology Services, Italy) and given the code VIP-1. The Ovation^®^ Ultralow V2 DNA-Seq Library Preparation Kit (NUGEN, San Carlos, CA) was used to prepare the library according to the manufacturer’s instructions. Both the input and final libraries were quantified using a Qubit 2.0 fluorometer (Invitrogen, Carlsbad, CA), and the quality was checked using an Agilent 2100 Bioanalyzer High Sensitivity DNA assay (Agilent Technologies, Santa Clara, CA). Libraries were then prepared for sequencing and sequenced on an Illumina NovaSeq 6000 platform (Illumina, San Diego, CA, USA) in paired-end 150 mode, which produced 51,327,588 reads per sample.

### Reads preprocessing and assembly

The quality-based filtration of the reads was carried out using Trimmomatic version 0.39 [24] with Illumina adaptor clipping option, sliding window trimming of a minimum of 4 bases, and average required quality of 20. Subsequently, filtered reads were assembled using the Unicycler version 0.4.8 assembler [25] on the BV-BRC server (https://www.bv-brc.org) accessed on August 3, 2024.

### VIP-1 strain typing and phylogeny

A phylogenomic-based genome typing of the isolate VIP-1 was performed using the Type Strain Genome Server (TYGS) [26] and the GTDB-Tk version 2.1.0 toolkit against the release 220 (28/10/2024) of the Genome Taxonomy Database (GTDB) [27]. The average nucleotide identity (ANI) and digital DNA–DNA hybridization (dDDH) between the genome of the isolate VIP-1 and its most related type strains were calculated using the JSpecies server [28] and Genome-to-Genome Distance Calculator [29]. The average amino acids identity (AAI) was calculated using the AAI calculator tool [30]. Additionally, multilocus sequence analysis (MLSA) was performed based on concatenated sequences of six housekeeping genes: 16S rRNA (∼1400 bp), *atpD* (∼1450 bp), *gyrB* (∼2100 bp), *recA* (∼1100 bp), *rpoB* (∼3500 bp), and *trpB* (∼1250 bp). The MLSA distance was calculated using MEGA11 software [31].

### Reference-guided scaffolding and VIP-1 genome annotation

RagTag version 2.1.0 was used for reference-guided scaffolding using the default parameters [32]. The Rapid Annotations using Subsystems Technology (RAST) [33], Prokka [34], and Bakta [35] were used to annotate the VIP-1 genome. To comprehensively identify potential antibiotic resistance genes (ARGs), we employed multiple tools, including the Resistance Gene Identifier (RGI 6.0.3) and the Comprehensive Antibiotic Resistance Database (CARD 4.0.0) [36], ABRicate version 1.0.1 [37], and AMRFinderPlus version 3.12.8 [38]. The mobileOG-db version 1.1.3 was used to annotate the bacterial mobile genetic elements (MGEs) [39]. IslandViewer 4 was used to analyze the genomic islands (GIs) within the genome of VIP-1 [40]. To investigate the main metabolic processes in the strain VIP-1, KEGG pathway annotation and reconstruction were performed using GhostKOALA, which automatically assigned KEGG Orthology (KO) identifiers and mapped the predicted protein products from the coding sequences (CDSs) of the strain VIP-1 to metabolic pathways [41]. The P2CS database was used to annotate the two-component systems (TCSs) within the genome of VIP-1 [42]. The Cluster of Orthologous Genes (COG) database was used to classify VIP-1 protein sequences into the different COG functional categories [43]. DIAMOND [44] was used to align the predicted protein sequences against the Carbohydrate-Active enZymes Database (CAZy) [45] to identify genes encoding enzymes involved in carbohydrate metabolism. The BGCs responsible for secondary metabolites production and their similarities to known clusters were identified using antiSMASH bacterial version 6.0 [46].

### Comparative genome analysis

A comparative genome analysis was conducted between the strain VIP-1 and other closely related *S. albidoflavus* strains isolated from marine and terrestrial environments to investigate genomic similarities and differences, particularly those potentially related to biosynthetic potential. Whole-genome alignments were performed using the MAUVE version 2.4.0 [47] to assess genomic rearrangements and conserved regions. OrthoVenn3 was used for the orthologous protein cluster analysis [48]. Additionally, principal component analysis (PCA) was performed using the ClustVis web server [49] based on the number of genes annotated under each COG functional category. Differences in the number, length, and types of BGCs were identified and compared using antiSMASH bacterial version 6.0. A similarity network analysis of the identified BGCs within each genome in the comparative study and all reference BGCs in the MIBiG version 3.1 database [50] was carried out using the Biosynthetic Gene Similarity Clustering and Prospecting Engine (BiG-SCAPE) version 1.1.9 on cut-off value of 0.5 [51].

## Results

### VIP-1 antimicrobial and antitumor activities

The metabolic extract of the strain VIP-1 showed antimicrobial activities against *S. aureus*,* P. aeruginosa*, and *C. albicans*(Table 1).

**Table 1:** Antimicrobial activity of *Streptomyces albidoflavus* VIP-1 metabolic extract against indicator bacterial and yeast strains. Values represent the diameter of inhibition zones in millimeters (mm)

	*Staphylococcus aureus*	*Escherichia coli*	*Pseudomonas aeruginosa*	*Candida albicans*
VIP-1 metabolic extract	15	0	20	17
Nystatin	-	-	-	30
Ampicillin	30	-	-	-
Imipenem	-	29	27	
Ceftazidime	-	28	20	-

**Fig. 2:**
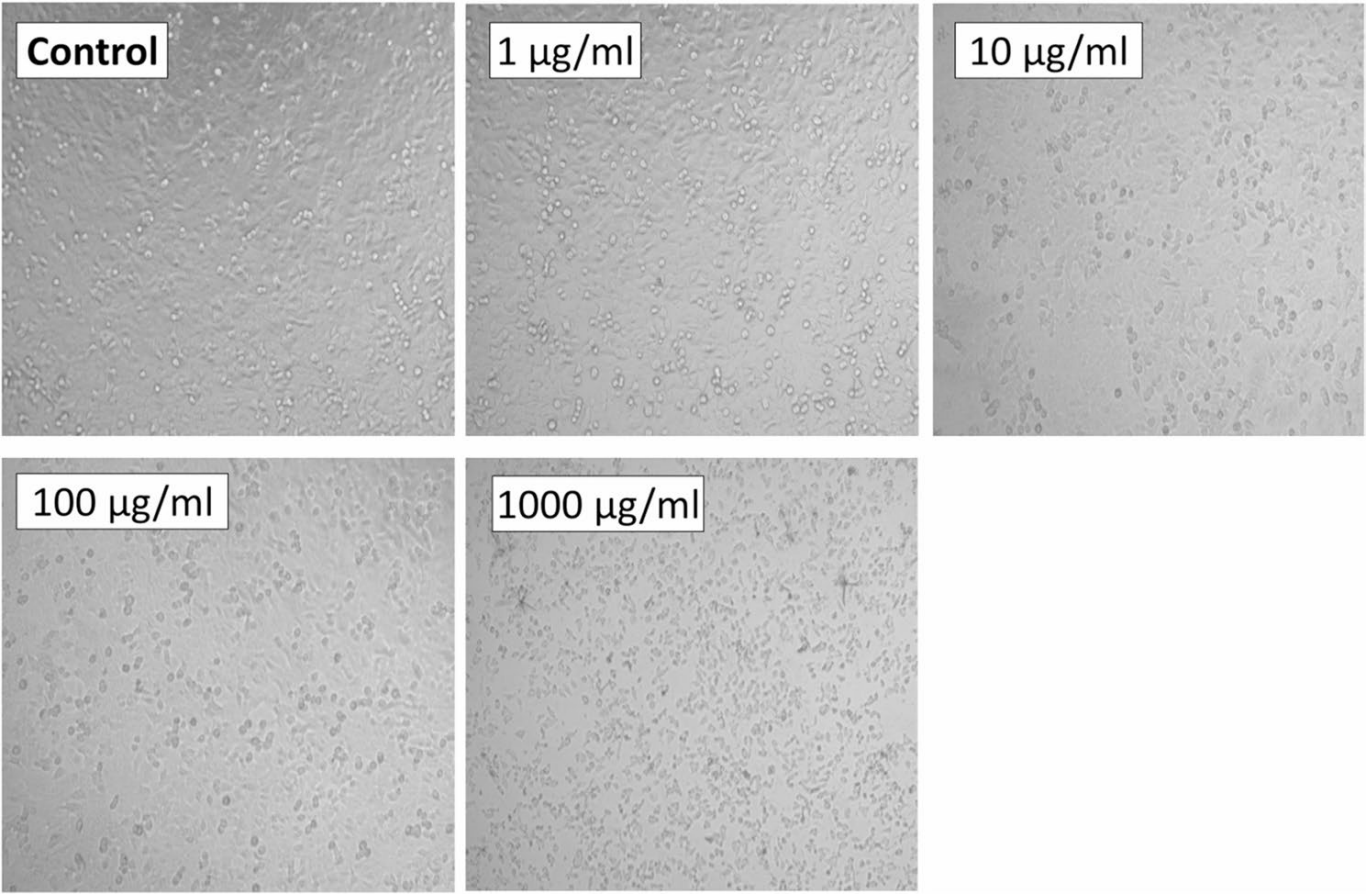
VIP-1 metabolic extract effect on lung cancer cells. Morphological changes observed in the lung cancer (A549) cell line after treatment with different concentrations of the VIP-1 metabolic extract, in comparison to a control

In the antitumor activity assay, the metabolic extract showed IC_50_ values of 85.836 µM and 136.642 µM against the A549 and HepG2 cancer cell lines, respectively. Notably, the IC_50_ values were lower than those of the DOX control, which showed IC_50_ values of 151.91 µM and 620.07 µM against the A549 and HepG2 cancer cell lines, respectively. This indicates that the VIP-1 metabolic extract has a higher cytotoxic activity, particularly in lung cancer cell lines (Fig. 2).

### VIP-1 genome characteristics and strain typing

**Fig. 3:**
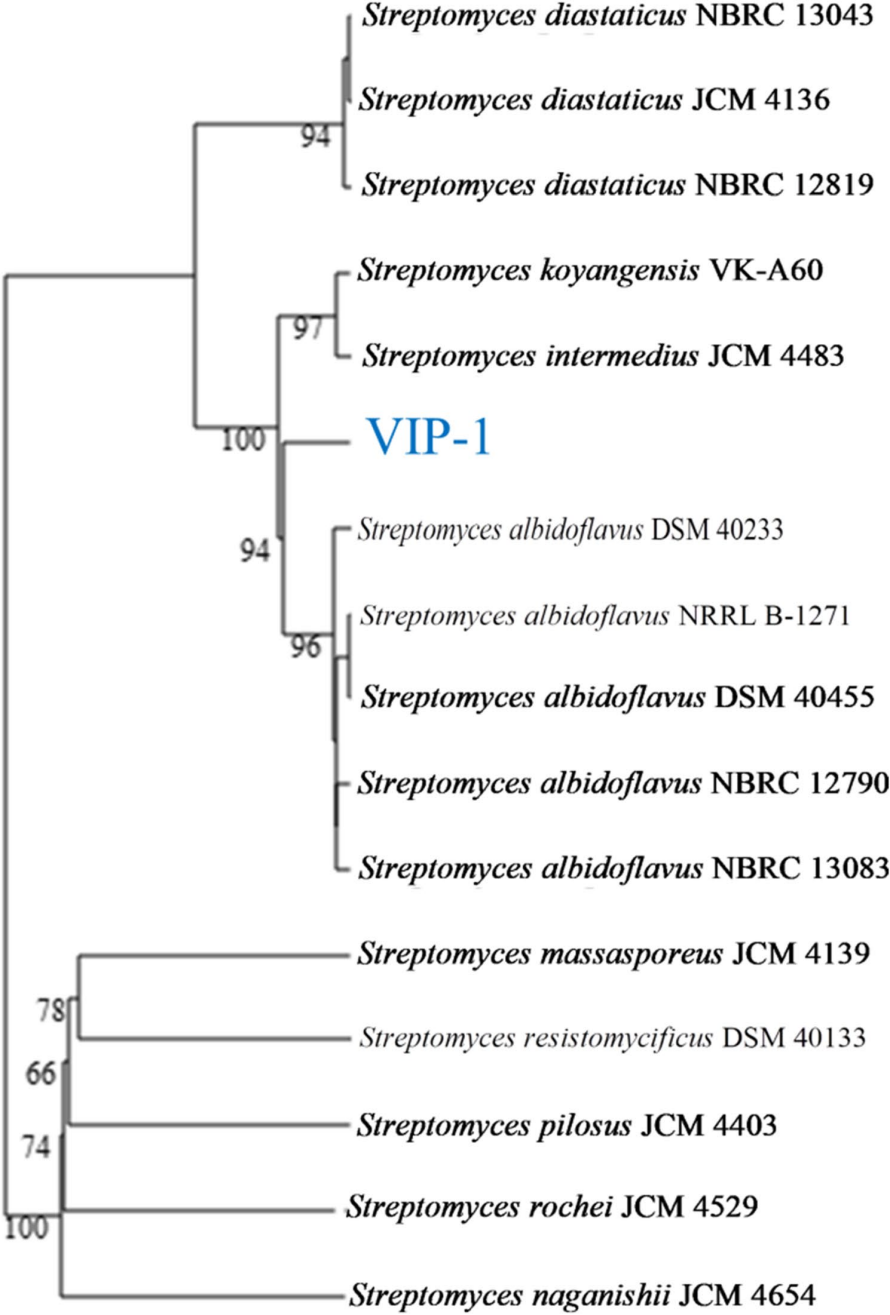
Phylogenomic tree of *Streptomyces albidoflavus* VIP-1. A phylogenomic tree constructed by the Type Strain Genome Server (TYGS) based on the genome of *S. albidoflavus* VIP-1 and its top-related type strains. Confidence values are displayed near the nodes. The taxonomic status of these type strains were checked and corrected according to the List of Prokaryotic Names with Standing in Nomenclature (www.lpsn.dsmz.de)

The draft genome of VIP-1 consisted of 87 contigs with N_50_ of 275,236 bp and a high GC content (73.54%). The total genome length was 7,090,100 bp. To determine the taxonomic affiliation of the isolate VIP-1, we performed a phylogenomic analysis using the GTDB-Tk toolkit. The GTDB-Tk classification assigned VIP-1 to the domain Bacteria, phylum Actinomycetota, class Actinomycetes, order Streptomycetales, family Streptomycetaceae, genus *Streptomyces*, and species *Streptomyces albidoflavus*. This classification was further supported by genome-based pairwise comparisons with closely related type strains using dDDH, AAI, and ANI (Table 2). A phylogenomic-based tree constructed using the TYGS server for the VIP-1 strain and its closely related type strains is shown in Fig. 3.

**Table 2:** Genomic relatedness of *Streptomyces albidoflavus* VIP-1 to closely related strains

Subject strain	Accession Number	dDDH (%)	∆GC (%)	ANIm (%)	ANIb (%)	AAI (%)
*Streptomyces albidoflavus* NRRL B-1271^T^	GCF_000719955.1	65.2	0.1	96.12	95.41	95.74
*Streptomyces albidoflavus* DSM 40,455 ^T^	GCF_004195735.1	64.90	0.09	96.11	95.42	95.59
*Streptomyces albidoflavus* NBRC 13,083 ^T^	GCF_004195755.1	64.70	0.05	96.1	95.37	95.66
*Streptomyces albidoflavus* DSM 40,233 ^T^	GCF_004195775.1	64.60	0.12	96.09	95.33	95.52
*Streptomyces koyangensis* VK-A60 ^T^	GCF_003428925.1	64.20	0.50	96.00	95.04	95.54
*Streptomyces diastaticus* JCM 4136 ^T^	GCF_014648955.1	41.30	0.12	91.65	90.92	90.94
*Streptomyces diastaticus* NBRC 13,043 ^T^	GCF_011170145.1	41.30	0.05	91.67	90.97	90.97
*Streptomyces diastaticus* NBRC 12,819 ^T^	GCF_011170105.1	41.10	0.01	91.59	90.83	90.88
*Streptomyces rochei* JCM 4529 ^T^	GCF_014650215.1	22.50	1.06	85.47	77.55	73.40
*Streptomyces naganishii* JCM 4654 ^T^	GCF_014650575.1	22.40	0.91	85.49	77.23	73.09
*Streptomyces resistomycificus* DSM 40,133 ^T^	GCF_001514265.1	22.30	2.48	85.24	76.33	72.66
*Streptomyces massasporeus* JCM 4139 ^T^	GCF_014648995.1	22.30	2.22	85.29	76.2	72.60
*Streptomyces pilosus* JCM 4403 ^T^	GCF_014649835.1	22.30	1.05	85.29	77.38	73.12
*Streptomyces albidoflavus* RKJM-0023	GCA_038242915.1	64.90	0.15	96.08	95.55	95.76
*Streptomyces albidoflavus* W68	GCF_015601545.1	94.70	0.16	99.56	99.44	99.30

*Digital DNA-DNA hybridization (dDDH), the difference in GC content (∆GC), the average amino acids identity (AAI), and the average nucleotide identity (ANI). The taxonomic status of these strains was checked and corrected according to the List of Prokaryotic Names with Standing in Nomenclature (www.lpsn.dsmz.de). The (T) letter represents type strains

VIP-1 showed the highest dDDH (65.2%), ANIm (96.12%), and AAI (95.74%) values with the type strain *S. albidoflavus* NRRL B-1271. The MLSA distance between the two genomes was found to be 0.00843, which can be considered within the gray zone for species delineation (between 0.008 and 0.014) as defined by Hu et al. (2022), making the ANIm and dDDH values essential confirmatory measures for taxonomic classification [52]. Although the values of dDDH below 70% are accepted for species delineation within the genus of *Streptomyces*, the ANIm and AAI values were found to be above the 95% cut-offs for species delineation, suggesting they are the same species [52]. Furthermore, the non-type strain *S. albidoflavus* W68 exhibited the closest genomic relationship to VIP-1, with ANIm, AAI, and dDDH values of 99.56%, 99.30%, and 94.70%, respectively. Collectively, these results support that strain VIP-1 belongs to the species *S. albidoflavus.*

### *S. albidoflavus *VIP-1 genome annotation and genome mapping

**Fig. 4:**
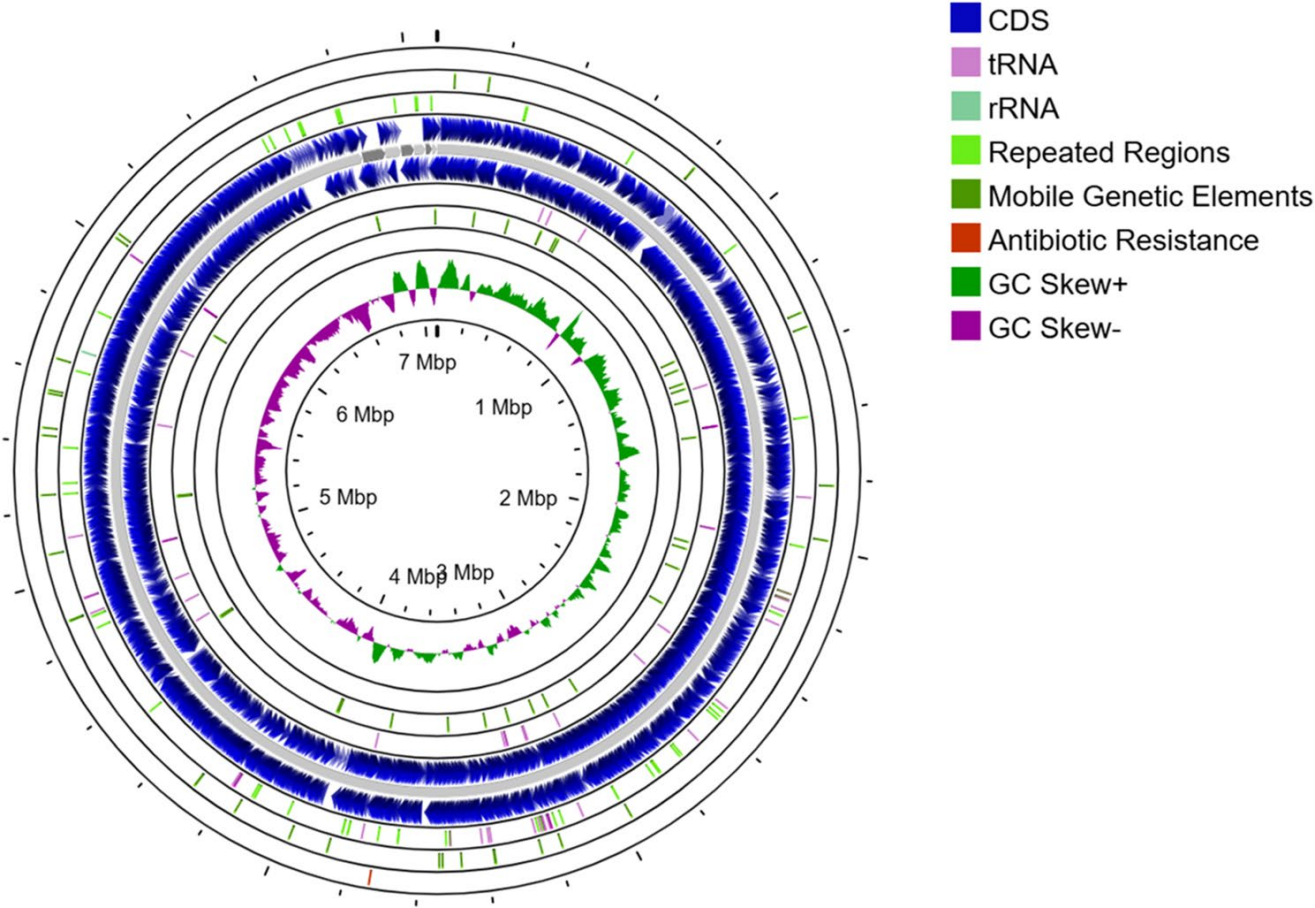
Genome map of *Streptomyces albidoflavus* VIP-1. A circular diagram illustrating the genome map of the strain VIP-1, visualized using the Proksee server (accessed on March 10^th,^ 2024). The gray circle represents the seven scaffolds. CDS, tRNA, rRNA, and repeated regions were located based on RAST annotations. The Comprehensive Antibiotic Resistance Database (CARD) was used to identify antimicrobial resistance-related genes, and the mobileOG-db was used to annotate genes related to mobile genetic elements

The reference-guided scaffolding of the VIP-1 draft genome resulted in 7 scaffolds. The largest was 6,825,844 bp in length, and the smallest was 17,715 bp in length. The genome map of *S. albidoflavus* VIP-1 is shown in Fig. 4.

The RAST, Prokka, and Bakta pipelines were used to annotate the genome of *S. albidoflavus *VIP-1. Notably, Bakta and RAST returned more annotations with functional assignments (Table 3).

**Table 3:** Summary of *Streptomyces albidoflavus* VIP-1 genome annotation results

	RAST	Prokka	Bakta
Total CDs	6,182	5,889	5,896
CDs with functional annotations	4,183	3,052	5,765
Hypothetical CDs	1,999	2,837	131
rRNA	1	3	3
tRNA	65	82	68

*S. albidoflavus* VIP-1 genome was analyzed for potential ARGs using CARD-RGI, AMRFinderPlus, and ABRicate. This integrated analysis revealed the presence of seven ARGs (Table 4).

**Table 4:** Summary of antibiotic resistance genes identified in *Streptomyces albidoflavus* VIP-1 using different bioinformatics tools

Gene	Product	Antibiotic Class	Identity	Tool
*rox*	Rifampin monooxygenase	Rifamycin	73.00%	CARD-RGI
*HelR*	Helicase-like RNA polymerase protection protein	Rifamycin	71.70%	CARD-RGI
*bla2a*	Exo family class A beta-lactamase	Beta-lactams	94.59%	AMRFinderPlus
*catA*	Type A-5 chloramphenicol O-acetyltransferase	Phenicol	93.64%	AMRFinderPlus
*aac(6’)*	Aminoglycoside 6’-N-acetyltransferase	Aminoglycosides	99.38%	AMRFinderPlus
*aph(3’’)-Ia*	Aminoglycoside phosphotransferase	Aminoglycosides	80.26%	ABRicate (ResFinder)
*aac(6’)-Isa*	Aminoglycoside 6’-N-acetyltransferase	Aminoglycosides	80.62%	ABRicate (ResFinder)

The presence of diverse MGEs indicates a genomically dynamic strain with potential for horizontal gene transfer, genomic rearrangements, and phage-mediated regulation, which enhances the adaptation to the marine environment. A total of 67 MGE-related genes were annotated using the mobileOG-db database. The highest number of annotations was in the categories of replication, recombination, and repair, while the smallest number was related to the stability, transfer, and defense categories (Supplementary Table S1). Additionally, the GIs analysis indicated the presence of 42 GIs of variable sizes distributed over the genome of *S. albidoflavus* VIP-1 (Supplementary Fig. S1). These islands harbored 266 CDSs, with only 110 of these CDSs annotated as functional proteins (Supplementary Table S2).

To better understand the ecological adaptations of *S. albidoflavus* VIP-1 to the marine environment, we investigated genes related to various stress responses and environmental challenges commonly encountered in marine ecosystems. These challenges include unusual temperature, low pH, osmotic- and oxidative stress, pressure, and nutrient starvation. Several genes related to stress adaptation, including antioxidant enzymes, osmoprotectant transporters, DNA repair proteins, molecular chaperones, and efflux systems were identified (Supplementary Table S3). These genetic elements are believed to play a crucial role in enabling the strain to thrive under the extreme and dynamic conditions of the Red Sea.

Two-component systems (TCSs) are essential for bacterial survival and adaptation to various environmental stresses. These systems enable bacteria to sense changes in their surroundings and respond accordingly, ensuring their ability to thrive in diverse conditions [53, 54]. In total, 136 TCS-related genes were detected within the genome of *S. albidoflavus* VIP-1. The products of these genes were classified into 72 histidine kinases (49 classic, 1 hybrid, and 22 incomplete), 63 response regulators, and a phosphotransfer protein.

**Fig. 5:**
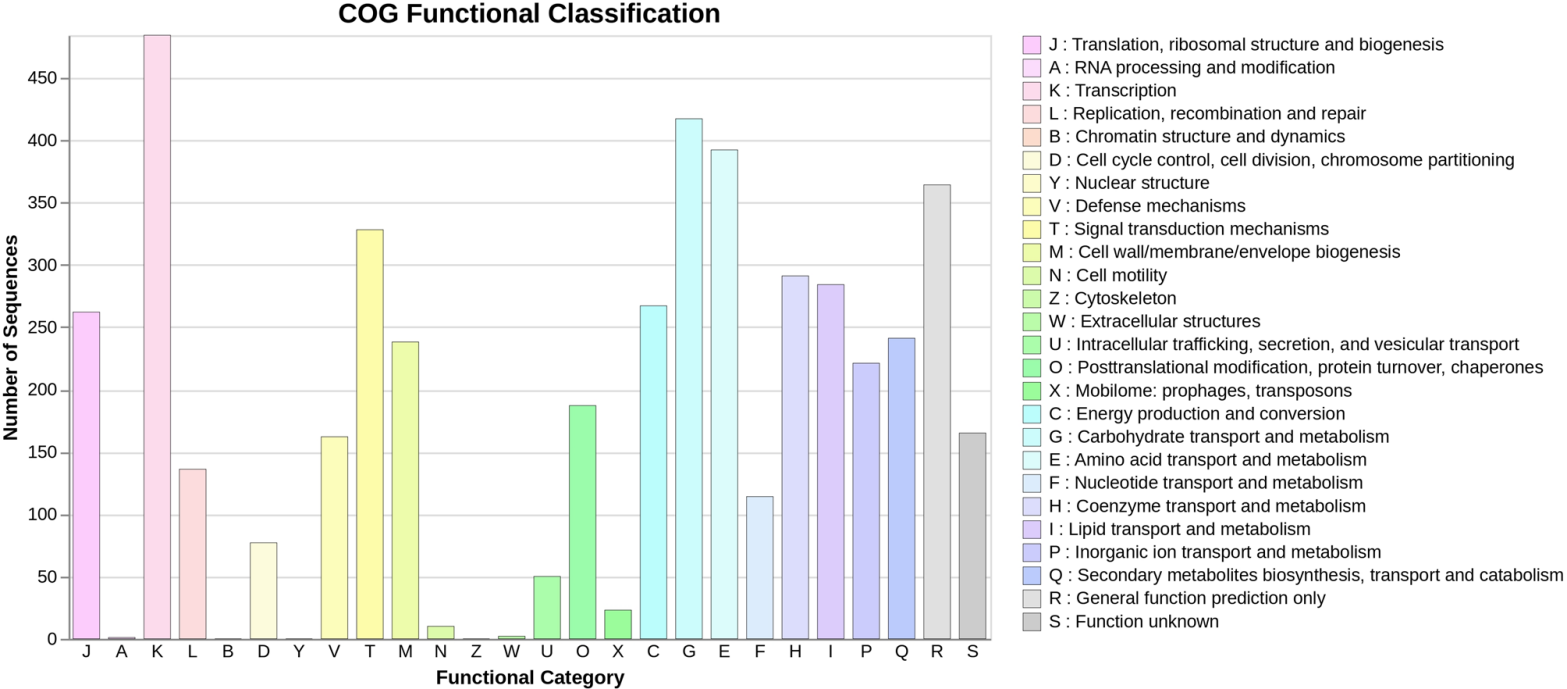
Functional protein classification of *Streptomyces albidoflavus* VIP-1. A bar chart showing the classification of proteins encoded by the *S. albidoflavus* VIP-1 genome into Clusters of Orthologous Genes (COG) functional categories

Approximately 75.60% of the coding genes within the genome of *S. albidoflavus* VIP-1 were assigned to a COG functional category, with 5.11% related to secondary metabolites biosynthesis, transport, and catabolism (Fig. 5). Out of the 6,182 CDSs detected within the genome of *S. albidoflavus* VIP-1, 2,372 received KO annotations. Additionally, three complete KEGG modules responsible for the biosynthesis of terpenoids and polyketides were detected within the genome of *S. albidoflavus* VIP-1, including the non-mevalonate pathway for C5 isoprenoid biosynthesis, the C10-C20 isoprenoid biosynthesis in bacteria, and the C10-C20 isoprenoid biosynthesis in archaea. KEGG annotation revealed the presence of 43 genes involved in terpenoid and polyketide metabolism, in addition to 49 other genes linked to the biosynthesis of additional secondary metabolites.

A total of 141 CAZyme-coding genes classified into five CAZymes classes were identified within the genome of *S. albidoflavus* VIP-1 (Table 5). This represents approximately 2.3% of the total predicted CDSs. In addition to catalytic CAZymes, there were 33 carbohydrate-binding modules (CBMs), which are non-catalytic domains commonly associated with CAZymes and facilitate substrate recognition and binding. The most representative class was glycoside hydrolase (GH), accounting for 50.35% of all CAZyme-coding genes. In contrast, the least representative class was polysaccharide lyase (PL), which makes up only 0.71% of the CAZyme repertoire.

**Table 5:** Classification and abundance of CAZyme-coding genes in the genome of *Streptomyces albidoflavus* VIP-1

Class	Gene type (number of genes)
Auxiliary activities	AA10 (3), AA3(1)
Carbohydrate esterases	CE1 (1), CE14 (2), CE4 (5), CE7 (1), CE9 (1)
Glycoside hydrolase	GH0 (5), GH1 (5), GH114 (1), GH13 (11), GH15 (2), GH16 (1), GH171 (1), GH18 (4), GH183 (1), GH184 (2) GH19 (3), GH2 (1), GH20 (2), GH23 (1), GH25 (3), GH3 (5), GH31 (1), GH32 (2), GH33 (1), GH39 (1), GH4 (2), others (16)
Glycosyl-transferases	GT0 (3), GT1 (5), GT2 (19), GT20 (1), GT28 (3), GT4 (11), Others (13)
Polysaccharide lyases	PL31 (1)

### Biosynthetic gene clusters (BGCs) in *S. albidoflavus* VIP-1

*Streptomyces* is known for having a large number of BGCs of various types, including PKS, NRPS, lanthipeptide, and terpene BGCs [55]. A total of 23 BGCs were detected by the antiSMASH 6.0 (Table 6). The BGC genomic content of *S. albidoflavus* VIP-1 was 1,106,046 bp, approximately 15.60% of the total genome size. Six BGCs identified with antiSMASH 6.0 showed 100% similarity to known BGCs from actinobacteria. Additionally, four BGCs showed similarities of 75% or higher to known BGCs. The remaining 13 BGCs are cryptic and show no significant similarity to any known BGCs.

**Table 6:** The biosynthetic gene clusters (BGCs) identified by antiSMASH 6.0 within the genome of *Streptomyces albidoflavus* VIP-1

Cluster	Type	Size (bp)	Most similar known cluster	Identity
1	terpene, NRPS-like, NRPS	76,572	valinomycin/montanastatin	13%
2	T1PKS, NRPS	49,411	SGR PTMs/SGR PTM Compound b/SGR PTM Compound c/SGR PTM Compound d	100%
3	Terpene	26,548	Hopene	76%
4	RiPP-like	10,216	hexacosalactone A	4%
5	RiPP-like	11,329		
6	NI-siderophore	33,076	Kanamycin	13%
7	Terpene	22,286	Geosmin	100%
8	Terpene	20,975	julichrome Q3-3/julichrome Q3-5	25%
9	thiopeptide, LAP, RRE-containing	34,699	fluostatins M-Q	4%
10	lanthipeptide-class-iii	22,580	SAL-2242	100%
11	NRPS	62,233	dechlorocuracomycin	16%
12	NRPS, LAP	108,844	surugamide A/surugamide D	95%
13	NI-siderophore	29,821	desferrioxamine B	100%
14	Ectoine	10,399	Ectoine	100%
15	RiPP-like, terpene	28,009	Isorenieratene	85%
16	RiPP-like	11,368	Streptamidine	75%
17	T3PKS, T1PKS, NRPS-like, NRPS, lanthipeptide-class-ii	295,391	Candicidin	100%
18	NRPS, T1PKS	81,224	Lankamycin	16%
19	T1PKS	55,980	quinolidomicin A	23%
20	T1PKS, NRPS	45,015	gargantulide B/gargantulide C	31%
21	T1PKS	40,386	Rosamicin	26%
22	T1PKS, terpene	22,895	Ossamycin	25%
23	Butyrolactone	6,789	griseoviridin/fijimycin A	5%

**Fig. 6:**
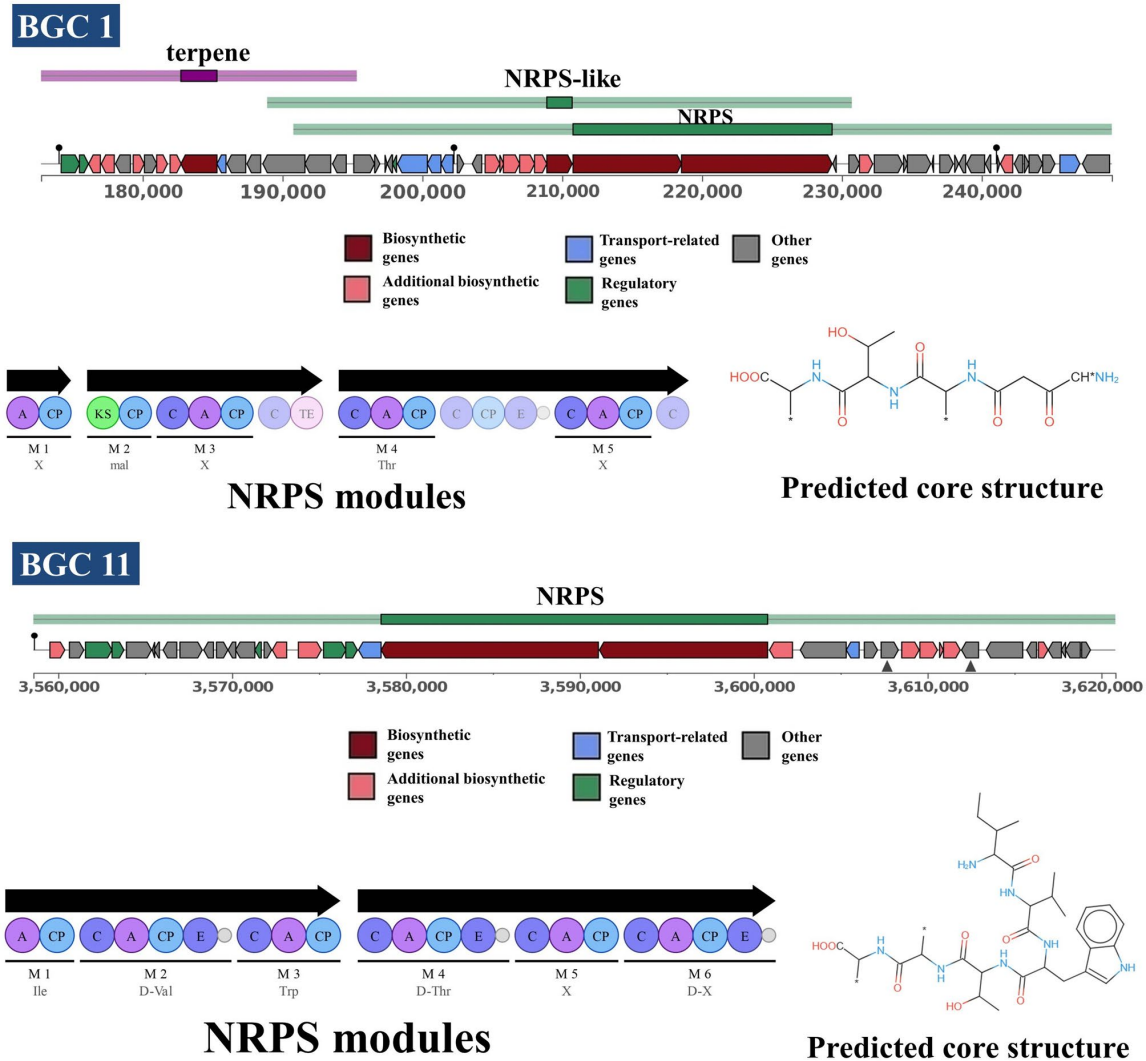
Biosynthetic gene clusters 1 and 11 identified within *Streptomyces albidoflavus* VIP-1 genome. The non-ribosomal peptide synthetase (NRPS) modules and predicted core scaffold structures of biosynthetic gene clusters (BGCs) 1 and 11, identified in the *S. albidoflavus* VIP-1 genome using antiSMASH 6.0

PKS and NRPS BGCs are often associated with bioactive compounds, particularly antimicrobial agents [12]. The genome of *S. albidoflavus* VIP-1 shows the presence of 10 PKS and/or NRPS-related BGCs. The NRPS clusters 1 and 11 showed no significant similarity with any known reference BGCs in the MIBiG database, suggesting that they may encode previously uncharacterized or novel bioactive compounds (Fig. 6). Clusters 18 to 23 may be incomplete BGCs due to their location at contig ends, potentially limiting our understanding of their full biosynthetic potential.

### Comparative genome analysis of *S. albidoflavus *VIP-1 and closely related strains

In this section, we compared the genome of *S. albidoflavus* VIP-1 with those of other marine- and terrestrial-derived strains from the species *S. albidoflavus* to identify both similarities and differences. The genomes included in this comparative analysis are listed in Table 7.

**Table 7:** Isolation sources and genome sizes of strains used in the comparative genomic study with *Streptomyces albidoflavus* VIP-1

Strain	Isolation Source	Genome Size (bp)
*S. albidoflavus* NRRL B-1271	Soil	7,084,693
*S. albidoflavus* DSM 40,455	Contaminated Plate	6,969,071
*S. albidoflavus* NBRC 13,083	Potato scab	7,042,329
*S. albidoflavus* RKJM-0023	*Halocynthia papillosa *(marine tunicate)	7,031,575
*S. albidoflavus* W68	Marine sediment	6,796,629

**Fig. 7:**
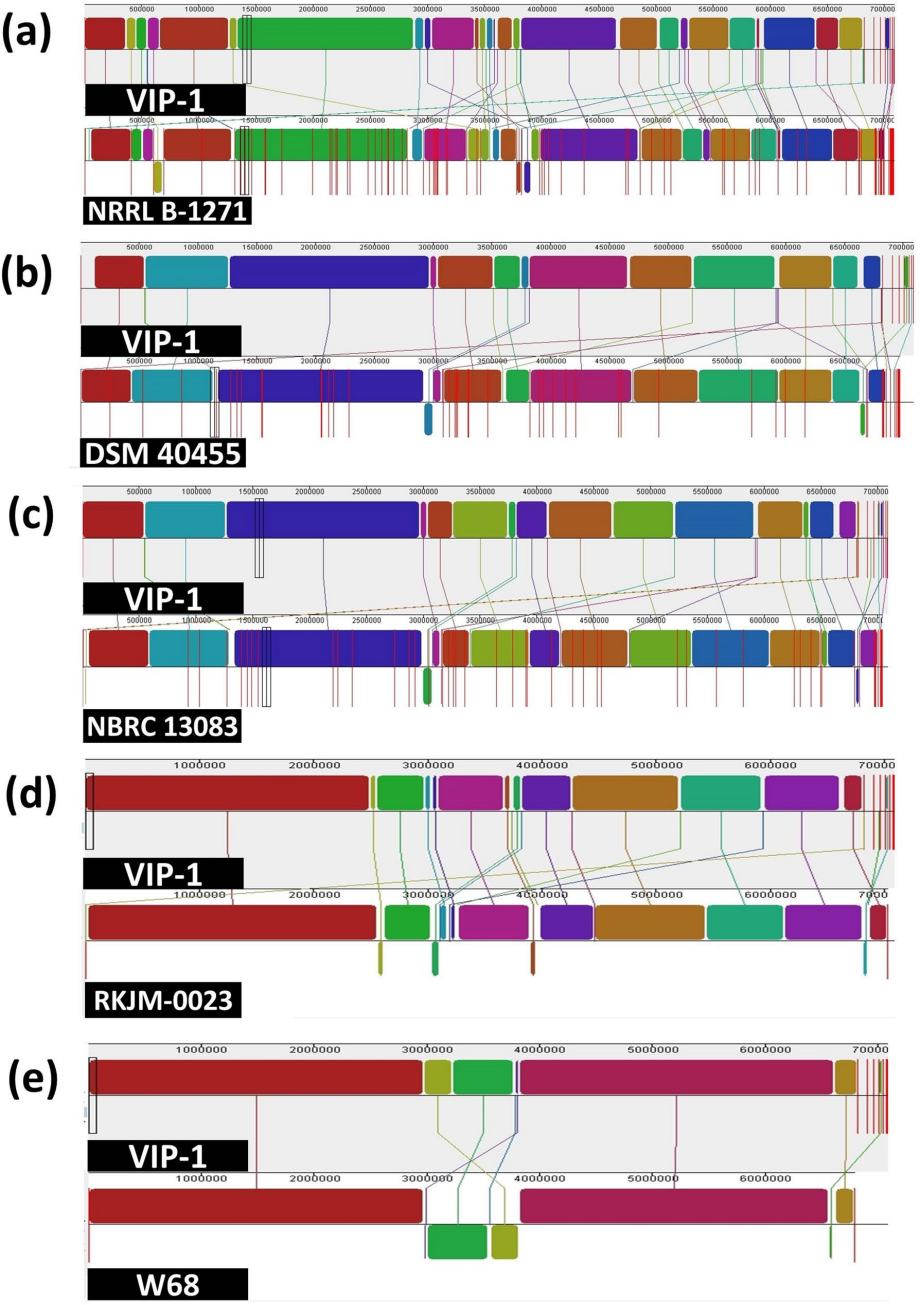
Genome alignment of *Streptomyces albidoflavus* VIP-1 and other related strains. A figure illustrating the results of genome alignment using MAUVE version 2.4.0 between the genome of *S. albidoflavus* VIP-1 (as a reference) and the genomes of *S. albidoflavus* NRRL B-1271 (**a**), *S. albidoflavus* DSM 40,455 (**b**), *S. albidoflavus* NBRC 13,083 (**c**), *S. albidoflavus* RKJM-0023 (**d**), and *S. albidoflavus* W68 (**e**)

The MAUVE genome alignment of *S. albidoflavus* VIP-1 with the five related strains revealed varying degrees of genomic conservation across the compared genomes (Fig. 7). In contrast to the terrestrial strains NRRL B-1271, DSM 40455, and NBRC 13083, comparisons between VIP-1 and the two marine-derived strains RKJM-0023 and W68 showed higher levels of synteny with fewer disruptions in locally collinear blocks (LCBs) order. Among the two, the strain W68 showed the highest genomic structural similarity to VIP-1, suggesting a closer evolutionary relationship.

**Fig. 8:**
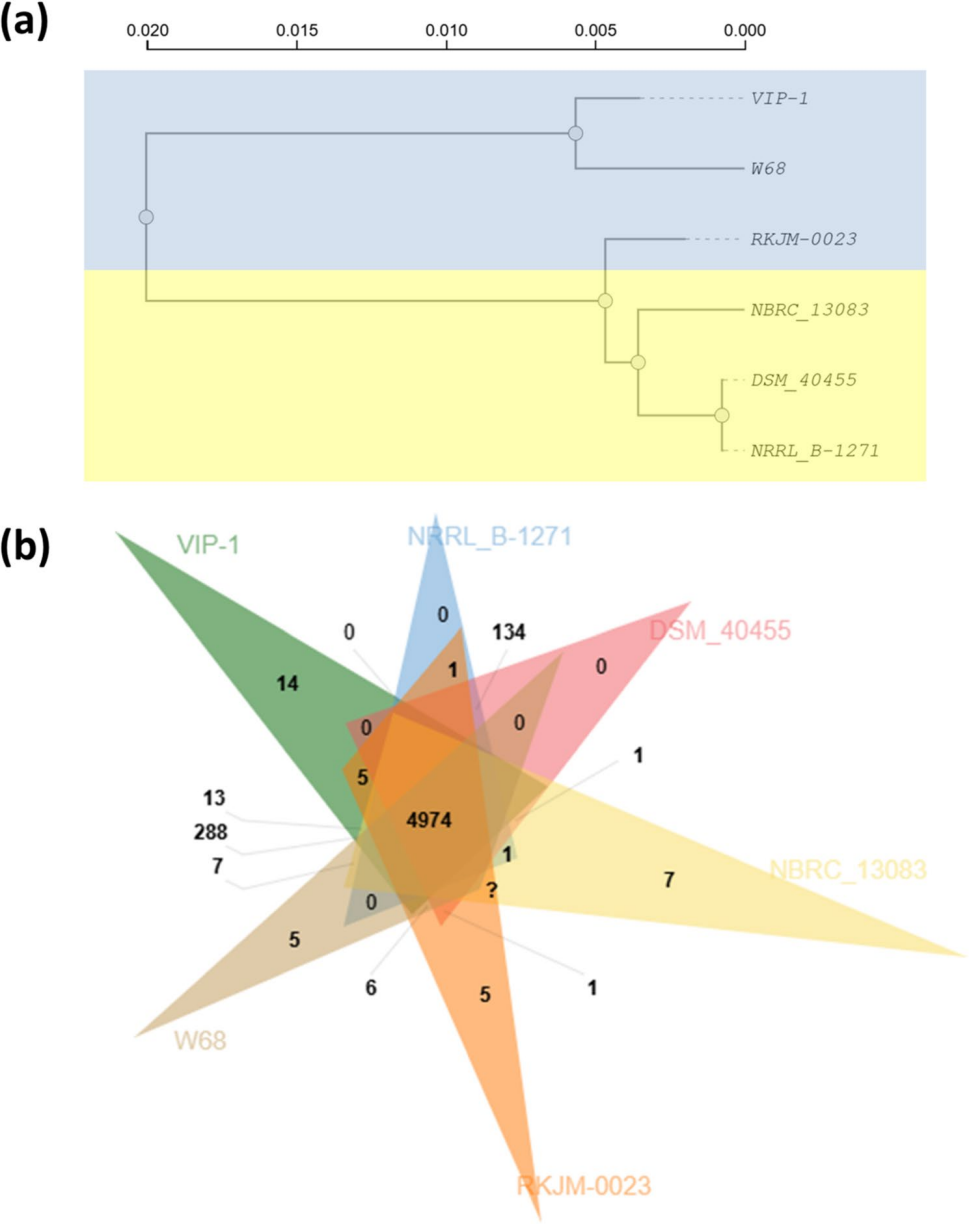
Orthologous protein clusters analysis of *Streptomyces albidoflavus* VIP-1 and other related strains. **a** A dendrogram constructed by OrthoVenn3 based on the identification of highly conserved single-copy genes. MUSCLE was used to perform sequence alignment. Trimal was used to extract and trim the conserved sequences. The Shimodaira-Hasegawa (SH) test method was used to assess the credibility of each phylogenetic node. **b** A Venn diagram indicates the number of shared orthologous protein clusters between the genomes of *S. albidoflavus* VIP-1 and other related *S. albidoflavus strains*

Similarly, the orthologous cluster profiles highlighted distinct evolutionary groupings among the isolates (Fig. 8a). The marine isolates VIP-1 and W68 formed a closely related clade. A second clade contained the terrestrial isolates, including NRRL B-1271, DSM 40455, and NBRC 13083, while the strain RKJM-0023 occupied an intermediate position between these two major clades, possibly reflecting a transitional lineage. The analysis revealed the presence of 4,974 orthologous protein clusters shared among all six genomes (Fig. 8b). However, each genome exhibited a variable number of unique orthologous clusters not found in any other strain. Notably, the marine isolate VIP-1 possessed 14 unique clusters, which is the highest among the other strains. Each of the other two marine-derived strains RKJM-0023 and W68, harbored five unique clusters. On the other hand, while the terrestrial strain NBRC 13083 harbored seven unique clusters, the other two terrestrial strains NRRL B-1271 and DSM 40455 had no unique clusters.

Gene Ontology (GO) annotation of VIP-1’s 14 unique clusters revealed their association with biologically relevant processes, including antibiotic biosynthesis, lipid metabolism, stress response mechanisms, and restriction-modification systems. Six of these clusters were associated with biosynthetic processes. These findings suggest that VIP-1 possesses additional genetic features that may support its survival in the marine environment and enhance its capacity for secondary metabolism.

Moreover, 288 orthologous protein clusters were exclusively shared between VIP-1 and W68. GO annotation of these 288 clusters revealed insights into their biological processes. The biological processes annotated within these clusters are related to metabolic processes, transport, stress response and regulation, and secondary metabolism. The high degree of overlap in these functional categories between VIP-1 and W68 underscores their close evolutionary relationship and probably exposure to similar ecological stressors.

**Fig. 9:**
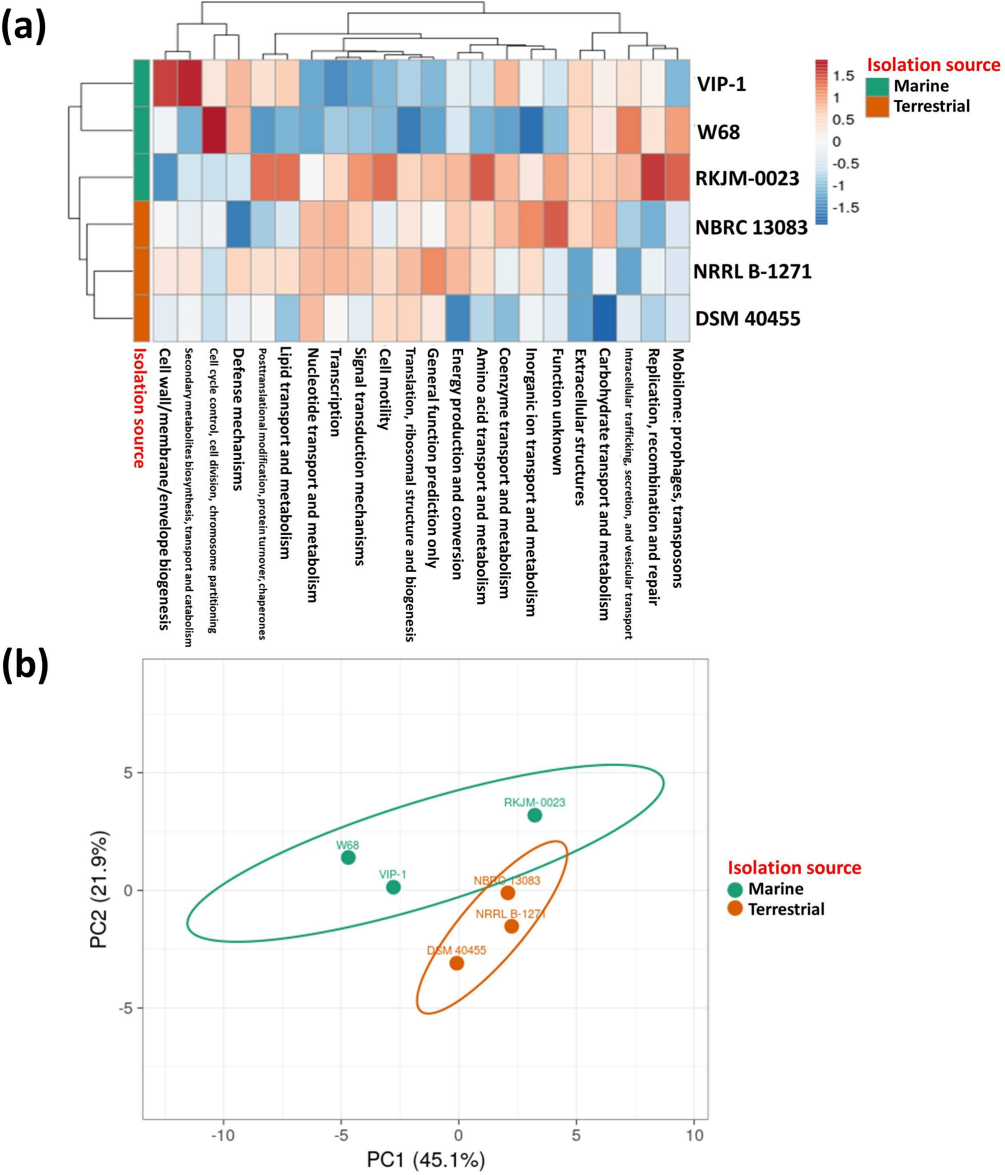
Clustering of *Streptomyces albidoflavus* VIP-1 and related strains based on their Orthologous Groups (COG) functional categories annotations. **a** A heatmap representing the number of annotations across the different Clusters of Orthologous Groups (COG) functional categories in *S. albidoflavus* VIP-1 and five other related *S. albidoflavus* strains. Unit variance scaling was applied to columns. Rows were clustered using Euclidean distance and average linkage. Columns were clustered using correlation distance and average linkage. **b** A Principal Component Analysis (PCA) plot of *S. albidoflavus* VIP-1 and five other *S. albidoflavus* strains based on their annotations across the different COG functional categories. The first two principal components (PC1 and PC2) account for 45.1% and 21.9% of the total variance, respectively. Ellipses represent 95% confidence intervals of belonging to the same cluster

By comparing the COG annotation profiles across the six strains, VIP-1 showed the highest number of annotations with the secondary metabolite biosynthesis and defense mechanisms categories (Fig. 9a), suggesting potentially enhanced capabilities in producing bioactive compounds and resisting harsh conditions. Notably, VIP-1 had a relatively lower count in the mobilome category (prophages and transposons) than the other marine strains, W68 and RKJM-0023. The PCA based on the COG annotation profiles of the six strains revealed observable distinctions between marine and terrestrial *S. albidoflavus* strains (Fig. 9b).

By comparing the occurrence of genes previously reported for their potential role in adaptation to the challenges found in the marine environment across the six strains, we observed significant variation in the occurrence of some genes. The Principal Component Analysis (PCA) of the gene occurrence data revealed a separation between marine and terrestrial strains (Supplementary Fig. S2a). Notably, certain genes such as *nuoJ *(NADH-quinone oxidoreductase subunit J) and *opuCD *(Glycine betaine/carnitine/choline transport system permease protein OpuCD) were completely absent from all terrestrial strains but were present in at least two of the marine strains. Additionally, genes like *yknY* (putative ABC transporter ATP-binding protein), *katE* (Catalase HPII), and *ydaD* (General stress protein 39) exhibited higher occurrence in the marine strains compared to their terrestrial counterparts (Supplementary Fig. S2b).

**Fig. 10:**
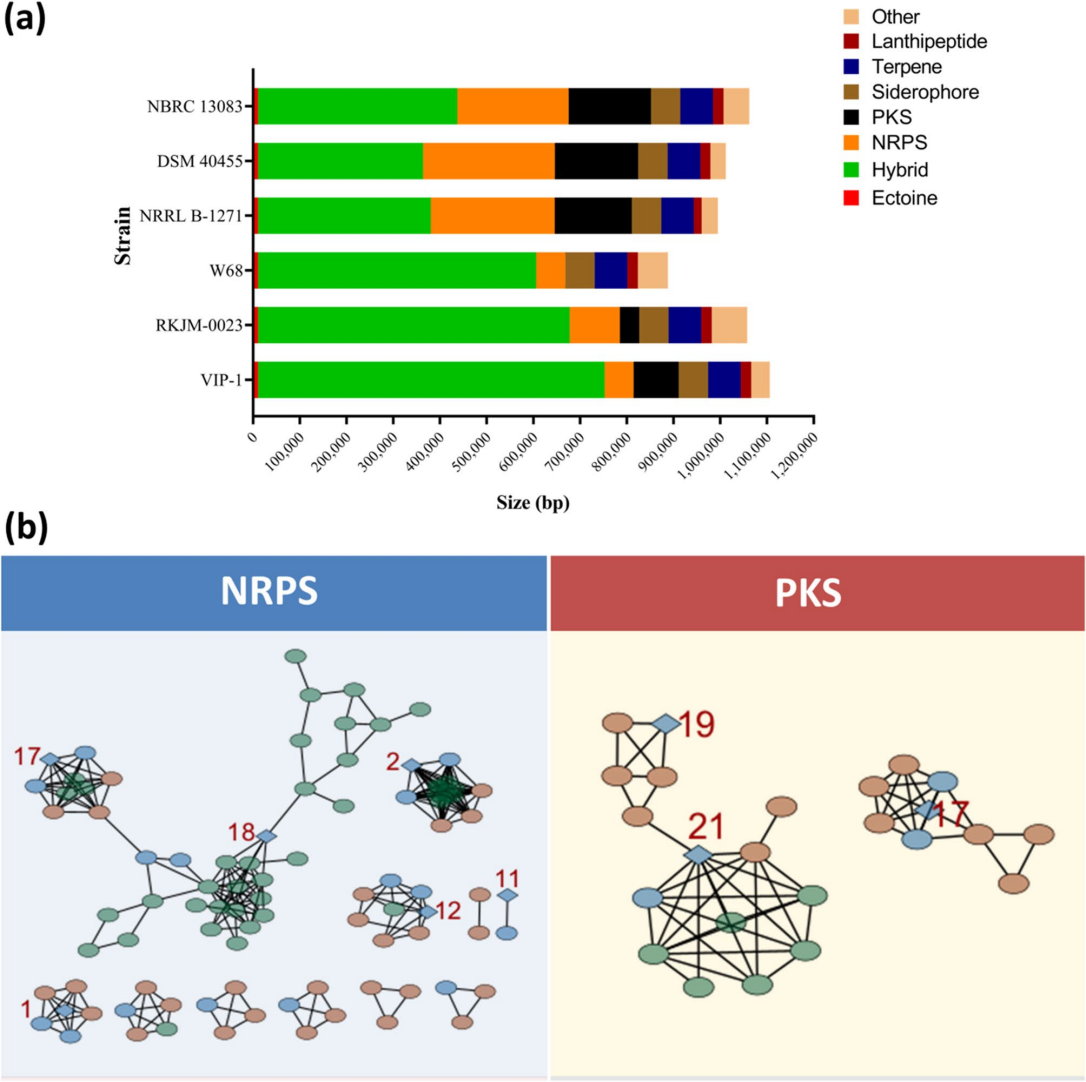
Comparison of biosynthetic gene clusters (BGCs) in *Streptomyces albidoflavus* VIP-1 and related strains (**a**) A bar chart indicates the differences in the total size per each type of BGCs in the genomes of *S. albidoflavus* VIP-1 and other related *S. albidoflavus* strains. (**b**) A network analysis of the polyketide synthases (PKSs) and non-ribosomal peptide synthetases (NRPSs) BGCs (excluding singletons) was carried out using BiG-SCAPE version 1.1.8 and visualized by Cytoscape version 3.10.3 for the BGCs detected using antiSMASH 6.0 within the genomes of *S. albidoflavus* VIP-1 and the other related *S. albidoflavus* strains. The brown nodes represent BGCs from terrestrial strains, while the blue ones represent those from marine strains. Diamond-shaped nodes represent BGCs from *S. albidoflavus* VIP-1, and the numbers in red color represent the cluster number within the genome of *S. albidoflavus* VIP-1

The comparative analysis of the BGC profiles across the six *S. albidoflavus* strains, considering total sizes and BGC types, revealed that VIP-1 exhibited an expanded repertoire of BGCs, evidenced by its larger total BGC size compared to the other strains (Fig. 10a). Additionally, the BiG-SCAPE network analysis of the BGCs from these six strains, alongside reference BGCs from the MIBiG database, showed varying levels of conservation and distinction among the different BGCs (Fig. 10b). For instance, the NRPS domains in clusters 2 (SGR PTMs), 12 (surugamides A-D), and 17 (candicidin) showed strong conservation across all six strains, with significant similarity to reference BGCs from the MIBiG database. In contrast, the NRPS domain in cluster 1 appeared conserved across the strains but lacked similarity to known MIBiG reference BGCs, even with a BiG-SCAPE cutoff of 0.5. Notably, the NRPS-type cluster 11 was unique to the marine strains VIP-1 and W68, suggesting a potentially distinct characteristic of these marine isolates. Furthermore, the Type-1 PKS clusters 19 and 21 showed high degrees of conservation across all six strains, with a degree of relatedness to reference BGCs from the MIBiG database for cluster 21. However, the PKS domain in cluster 17 exhibited no similarity to any known reference BGCs from the MIBiG database. Generally, when comparing the BGC profiles between marine and terrestrial strains, a general trend emerges. Marine isolates, including VIP-1, tend to harbor a larger total size of hybrid BGCs, which combine multiple biosynthetic modules. In contrast, terrestrial strains carry relatively higher total sizes of individual PKS and NRPS BGCs. This difference may reflect adaptations to their respective environments, with marine strains potentially relying on more complex, multifunctional BGCs.

## Discussion

Actinobacteria, particularly the genus *Streptomyces*, are well-known for their high ability to produce novel bioactive compounds [56]. Marine invertebrate-associated actinobacteria have been reported as active producers of secondary metabolites, especially those of antimicrobial activities. These microbial metabolites act as chemical defenses to protect the host from predators and pathogens [57]. In this study, we report the isolation of the marine bacterium *S. albidoflavus* VIP-1. The metabolic extract of this isolate exhibited broad-spectrum antibacterial activity against the Gram-positive *S. aureus* and Gram-negative *P. aeruginosa, *as well as potent antifungal properties against *C. albicans. *Furthermore, the metabolic extract of the isolated strain showed significant *in vitro* antitumor activity against human hepatocellular carcinoma and non-small cell lung adenocarcinoma cell lines. Based on its significant bioactivity, *S. albidoflavus* VIP-1 was considered a potentially promising source for novel drugs. To understand the underlying mechanisms of these bioactivities and the influence of the marine environment on the metabolic profile of *S. albidoflavus* VIP-1, we conducted a genome-wide analysis and comparative genomics study with its closest relatives from marine and terrestrial origins.

Genomic analysis confirmed that isolate VIP-1 belongs to the genus *Streptomyces*, particularly the species of *S. albidoflavus*. The phylogenomic analysis of VIP-1 and the type strain *S. albidoflavus* NRRL B-1271 showed high similarity but also revealed inconsistencies between different genomic markers. Specifically, while ANIm and AAI values exceeding 95-96% strongly suggested they belong to the same species, the dDDH value of less than 70% lies below the generally accepted thresholds for species delineation [52]. In contrast, comparisons with the marine non-type strain *S. albidoflavus* W68 showed ANI and AAI values greater than 99%, along with a dDDH value exceeding 94%, indicating a highly close genomic relationship. To further clarify the taxonomic status of VIP-1, a classification based on the relative evolutionary divergence (RED) and ANI criteria was conducted using GTDB-Tk against the GTDB database. This analysis confirmed the assignment of isolate VIP-1 to the species *S. albidoflavus.*

The genomes of marine *Streptomyces* have been reported to harbor marine adaptation genes (MAGs) that are largely absent in their closest terrestrial relatives, underscoring their potential importance in facilitating survival in marine ecosystems [21, 58]. For instance, Albuquerque et al. (2021) described several potential MAGs in marine *Streptomyces *isolates, including genes encoding ABC transporters, redox enzymes, Na⁺/H⁺ antiporters, and transcriptional regulators [58]. The *S. albidoflavus* VIP-1 genome carries several genes encoding redox enzymes such as catalases, alkyl hydroperoxide reductases, and superoxide dismutases. This potent antioxidant system likely contributes to the strain’s resistance against oxidative stress resulting from nutrient depletion and unfavorable environmental conditions [59, 60]. Furthermore, we identified over 40 genes associated with putative ABC and osmoprotectant transportation systems in the genome of VIP-1, along with genes that encode cold shock proteins. The presence of these genes potentially supports the strain’s adaptation to low temperatures and high salinity [61]. Additionally, the presence of genes involved in DNA repair, protein folding, and general stress response further highlights the strain’s capacity to adapt to various environmental challenges [62–64]. Moreover, the abundance of two-component systems, which act as sensory and regulatory systems, likely enhances the ability of *S. albidoflavus* VIP-1 to interact with its environment, and to survive in marine ecosystems [65, 66].

MGEs, including transposons, integrative and conjugative elements (ICEs), and prophages, play an essential role in horizontal gene transfer and bacterial genome plasticity and facilitate the acquisition of new adaptive traits [67]. The presence of different types of MGEs in the genome of *S. albidoflavus* VIP-1 highlights its genomic dynamism. Among the MGEs identified by the mobileOG-db database and potentially confer adaptive advantages to *S. albidoflavus *VIP-1 within the marine environment is the multidrug efflux pump (Tap), which could enhance resistance to environmental toxins [68]. In addition, genes code for molecular chaperones DnaK, ClpB, and ClpX, which likely enhance the strain's stress tolerance under fluctuating marine conditions [69, 70]. The DNA repair proteins RnhA, RecA, and LexA may also promote genome stability against environmental stressors [64, 69, 71].

GIs are considered MGEs that integrate into bacterial chromosomes [72]. While many GIs integrate permanently, some remain mobile [73]. These GIs typically contain functionally related gene clusters that significantly contribute to niche adaptation and phenotypic diversification [67, 73]. Within the genome of *S. albidoflavus* VIP-1, 42 GIs were identified, harboring 110 genes with known functions. The repertoire of genes found in these GIs provides valuable insights into the potential adaptation mechanisms acquired by this isolate. For example, antimicrobial resistance genes, such as *abaF*, and *tetC*, provide the strain with an extra genetic defense strategy against antimicrobial compounds [74, 75]. The presence of the *qacA* gene further equips the bacterium with resistance to environmental biocides and pollutants by encoding the QacA efflux pump, which reduces intracellular concentrations of cationic antiseptic agents and thereby decreases susceptibility [76]. Moreover, genes involved in nutrient acquisition and metabolism were identified within the GIs, such as *treC* and *tauD*. The *treC* gene encodes trehalose-6-phosphate hydrolase (classified within CAZyme family GH13, subfamily 29), a key enzyme in trehalose metabolism that functions both as an osmoprotectant and a carbon source in various bacteria [77]. This gene likely plays a crucial role in adaptation to the hyperosmotic marine environment. Additionally, the presence of the *sodF1* gene, encoding superoxide dismutase [Fe-Zn] 1, underscores the bacterium’s capacity for oxidative stress response, further supporting its survival in challenging conditions [78].

CAZymes facilitate the uptake of nutrients for bacteria from the surrounding environment. Moreover, some CAZymes give bacterial strains a competitive advantage against fungi by degrading their cell walls [45, 79]. The CAZyme repertoire of *S. albidoflavus* VIP-1 highlights its enzymatic capacity for degrading complex carbohydrates, including cellulose, hemicellulose, chitin, and starch. This enzymatic versatility supports nutrient acquisition in nutrient-poor marine environments and may contribute to antagonistic interactions with competing pathogenic microbes. These findings align with the well-documented role of *Streptomyces* species in nature [80]. For instance, GH13 was the most abundant GH family identified in the genome of the marine isolate. The predicted gene products were classified into eight distinct GH13 subfamilies (3, 9, 10, 11, 13, 16, 30, and 32). These subfamilies are associated with multiple enzymatic activities, including α-amylases, maltosyltransferases, glycogen debranching enzymes, maltooligosyltrehalose trehalohydrolases, and pullulanases [81–85]. These enzymes are known to act on a variety of α-1,4-linked glucan substrates, including amylose, amylopectin, starch, and glycogen, highlighting the functional diversity and substrate versatility of GH13 CAZymes within this organism [82, 83, 85–87]. The second most abundant family in the *S. albidoflavus* VIP-1 genome was the GH1. This family’s main activity in *Streptomyces* species is the β-glucosidase activity and its main substrate is cellulose [87, 88]. Both families were found in several *Streptomyces *species regardless of the isolation source [80–82, 85, 86].

A total of 23 BGCs were identified within the genome of *S. albidoflavus *VIP-1. These BGCs produce various secondary metabolites, including known compounds with antimicrobial and antitumor activities. Among the 23 BGCs, 10 clusters had either single or hybrid PKS and NRPS domains. For example, we were able to identify hybrid PKS-1/NRPS BGCs with high identity to known ones responsible for the production of antimicrobial metabolites, such as the polycyclic tetramate macrolactams, and candicidin [89, 90]. Polycyclic tetramate macrolactams are a group of potent and broad-spectrum antimicrobial agents, while candicidin is a polyene macrolide known for its antifungal properties [89, 90]. The presence of these two clusters may explain the antibacterial and antifungal properties of the strain’s metabolic extract.

Furthermore, among the known BGCs identified within the genome of *S. albidoflavus *VIP-1 was the surugamides cluster. The surugamides family, including surugamides and acyl-surugamides, has been reported to exhibit diverse bioactivities such as cytotoxic, anthelmintic, and antifungal activities [91, 92]. This also may rationalize the strain's cytotoxic and antifungal activities. Almeida et al. (2019) conducted a study comparing the production of surugamide A by the marine sponge isolate *Streptomyces sp.* SM17 and its terrestrial relative*S. albidoflavus* J1074 [93]. The results showed differential production of surugamide A between the two closely related isolates, with SM17 producing higher levels of the compound than *S. albidoflavus* J1074 under all tested conditions [93].

A BGC of desferrioxamine B was also identified in the genome of *S. albidoflavus *VIP-1. Desferrioxamin B is a hydroxamate iron chelator, that plays an essential role in iron acquisition from the environment and is widely represented in the genus *Streptomyces* [94]. In addition to the above, we could identify a geosmin BGC in the genome of VIP-1. Geosmin is an earthy-smelling compound produced in aquatic ecosystems, mainly from cyano- and actinobacteria [95, 96]. Interestingly, some whole clusters showed no similarity to any known BGCs, suggesting that they may encode novel secondary metabolites, such as the NRPS clusters 1 and 11.

The comparative genome analysis between VIP-1 and five of its closest relatives from marine and terrestrial origins indicated significant differences between the selected marine and terrestrial strains. For example, the orthologous protein clusters analysis, supported by further analysis of COG functional annotations within the categories related to biosynthetic and defensive mechanisms, indicated that marine strains, including VIP-1, have extra unique genetic capabilities in these functions. Moreover, the BGC sequence similarity network analysis presented cluster 11 as a unique and exclusive BGC to marine isolates VIP-1 and W68. This cluster has not been found in the terrestrial strains or even clustered with any of the reference BGCs available on the MIBiG database. These observations agree with reports highlighting that marine *Streptomyces* species exhibit unique BGCs not found in their terrestrial counterparts [21, 97]. Interestingly, Xu et al. (2019) concluded that marine invertebrates-associated *Streptomyces* species carry more BGCs than sediment-driven ones, highlighting their considerable potential for secondary metabolite biosynthesis [98]. This agrees with our results as both tunicate-associated *S. albidoflavus* VIP-1 and RKJM-0023 have a higher number of BGCs and longer total BGCs size when compared to the sediment-driven strain W68. Interestingly, our comparative analysis revealed two potential MAGs present only in the marine strains, potentially conferring increased stress resistance. One is the *nuoJ* gene, a key gene in the *nuo*-operon that codes for Complex I of the electron transport chain [99]. This complex is essential for oxidative phosphorylation, facilitating ATP production by transferring electrons from NADH to quinone molecules. Previous studies presented the *nuo*-operon genes as potential MAGs, where the proton-pumping activity of Complex I helps maintain a proton gradient in seawater [21, 100]. A study by Ian et al. (2014) highlighted that marine-derived *Streptomyces* sp. SM17 and SM18 possess a partial *nuo*-operon, which was completely absent in their terrestrial relatives [21]. In our data, with the exception of the *nuoJ* gene, the same *nuo*-operon genes were found in the marine and terrestrial strains. The second one is the *opuCD* gene that encodes the permease component of the OpuC ABC transporter system, which is involved in the uptake of compatible solutes such as glycine betaine, carnitine, and choline [101]. These compounds are known as osmoprotectants that balance the internal osmotic pressure of bacteria without interfering with cell function. Marine-derived *Streptomyces* species were reported to accumulate these osmoprotectants intracellularly as an adaptive response to a high-osmolality environment [102].

## Conclusion

The genome analysis of *S. albidoflavus* VIP-1 highlights its ecological and biotechnological significance as a marine-derived actinobacterium. The strain showed strong antimicrobial and antitumor activities, supported by a diverse collection of BGCs, including those encoding NRPSs and PKSs. Comparative genomic analysis with other *S. albidoflavus* strains revealed that VIP-1 possesses an expanded biosynthetic potential, particularly when compared to terrestrial relatives. Genomic features such as stress response genes, compatible solute transporters, and two-component regulatory systems reflect potential adaptations to the marine environment. Overall, this study underscores the value of exploring symbiotic marine actinobacteria for novel bioactive compounds and provides a foundation for future investigations into the ecological roles and biosynthetic capabilities of underexplored marine *Streptomyces* species.

## Supplementary Information

Supplementary Material 1.

## Data Availability

The strain was deposited in EMCCN-NRC under accession number 3075. The datasets supporting this article’s conclusions are available in the National Centre for Biotechnology Information (NCBI) GenBank. Sequence information was made available to the NCBI BioProject accession PRJNA863669 and Genome accession GCA_024592655.1. https://www.ncbi.nlm.nih.gov/datasets/genome/?taxon=1886.

## Abbreviations

AAI: Average amino acids identity
ANI: Average nucleotide identity
ARGs: Antibiotic resistance genes
BGCs: Biosynthetic gene clusters
CAZy: Carbohydrate-Active enZymes Database
CBM: Carbohydrate-binding modules
dDDH: Digital DNA–DNA hybridization
DMSO: Dimethylsulfoxide
GI: Genomic island
GTDB: Genome Taxonomy Database
IC_50_: Half maximal inhibitory concentration
ICE: Integrative and conjugative elements
LCBs: Locally collinear blocks
MAG: Marine adaptation gene
MIBiG: Minimum Information about a Biosynthetic Gene cluster
MLSA: Multilocus sequence analysis
MTT: 3-(4,5-dimethylthiazol-2-yl)-2,5-diphenyltetrazolium bromide
NRPS: Non-ribosomal peptide synthetase
NSW: Natural seawater
PCA: Principal component analysis
PKS: Polyketide synthase
RAST: Rapid Annotations using Subsystems Technology
RED: Relative evolutionary divergence
RiPPs: Ribosomally synthesized and Post-translationally modified Peptides
R2A: Reasoner’s 2A agar
TCS: Two-component system
TYGS: Type Strain Genome Server
v/v: Volume per volume
