# Supplementary material for: Genomic insights into biosynthesis and adaptation in the bioactive marine bacterium Streptomyces albidoflavus VIP-1 from the Red Sea
Source: BMC Microbiol. 2025 Jun 26;25:372. doi: 10.1186/s12866-025-04109-x (PMC12199506; doi:10.1186/s12866-025-04109-x)

*Supplementary Material*

**Genomic Insights into Biosynthesis and Adaptation in the Bioactive Marine Bacterium *Streptomyces albidoflavus* VIP-1 from the Red Sea**

**Abdelrahman M. Sedeek^1,^ Hossam Elfeky^2^, Amro S. Hanora^2^, and Samar M. Solyman^2,3*^**

**Affiliations**

^1^Department of Microbiology & Immunology, Faculty of Pharmacy, Galala University, New Galala City, Suez 43511, Egypt

^2^Department of Microbiology & Immunology, Faculty of Pharmacy, Suez Canal University, Ismailia, Egypt

^3^Department of Microbiology & Immunology, Faculty of Pharmacy, Sinai University- Elkantara branches, Ismailia, Egypt

***Correspondence:**

Samar M. Solyman

[**Samar.mansour@su.edu.eg**](mailto:Samar.mansour@su.edu.eg)

**Table S1:** Summary of the mobile genetic elements-related genes identified within the *Streptomyces albidoflavus* VIP-1 genome

| **Category** | **Number of annotations** | **Key identified genes** |
| --- | --- | --- |
| Integration/excision | 12 | *insI3*, *pra1,* ISPlu7D_orfA, *tnpB*, tIS1421 |
| Replication/recombination/repair | 27 | *diaA, dnaB, dnaE, ftsK, ftsZ, gyrA, gyrB, nfi, nucS, radA, rarA, rnhA, recA, recQ, recR, rep, ruvB, rph, tag, topA, uvrA, uvrB, ung, xseB* |
| Phage | 19 | *clpB, clpP, dnaK, ftsH, lexA, nusA, whiB, clpX* |
| Stability/transfer/defense | 2 | SCO4629 |
| Transfer | 7 | *copR, oppF, ttrA, traSA1* |

**Table S2:** Genomic islands (GIs) identified in the *Streptomyces albidoflavus* VIP-1 genome using the IslandViewer 4 server, including the annotated genes within each GI (excluding hypothetical proteins).

| **Genomic Island Number** | **Size (bp)** | **Gene Name** | **Product** |
| --- | --- | --- | --- |
| 1 | 5140 | *ligB* | DNA ligase B |
| 2 | 5468 | *nagZ_1* | Beta-hexosaminidase |
|  |  | *rbsR_1* | Ribose operon repressor |
| 3 | 7640 | *lcfB_1* | Long-chain-fatty-acid--CoA ligase |
|  |  | *yfeW_2* | Putative D-alanyl-D-alanine carboxypeptidase |
|  |  | *rlmG* | Ribosomal RNA large subunit methyltransferase G |
| 4 | 5960 | *rnhA* | Ribonuclease H |
|  |  | *msuD* | Methanesulfonate monooxygenase |
| 6 | 5519 | *COQ5_1* | 2-methoxy-6-polyprenyl-1,4-benzoquinol methylase, mitochondrial |
| 7 | 14585 | *sprA* | Streptogrisin-A |
|  |  | *mdtD_1* | Putative multidrug resistance protein MdtD |
|  |  | *abaF_2* | Fosfomycin resistance protein AbaF |
|  |  | *thlA_1* | Acetyl-CoA acetyltransferase |
|  |  | *benM* | HTH-type transcriptional regulator BenM |
| 8 | 5777 | *lagD* | Lactococcin-G-processing and transport ATP-binding protein LagD |
|  |  | *btuD_2* | Vitamin B12 import ATP-binding protein BtuD |
|  |  | *slyA_2* | Transcriptional regulator SlyA |
| 9 | 4219 | *pobB_1* | Phenoxybenzoate dioxygenase subunit beta |
|  |  | *aruI* | putative 2-ketoarginine decarboxylase AruI |
|  |  | *bacC* | Dihydroanticapsin 7-dehydrogenase |
|  |  | *ped* | (S)-1-Phenylethanol dehydrogenase |
| 10 | 5096 | *ulaE* | L-ribulose-5-phosphate 3-epimerase UlaE |
|  |  | *cyoE* | Protoheme IX farnesyltransferase |
| 12 | 4819 | *tap_2* | Tripeptidyl aminopeptidase |
| 13 | 5355 | *gyrA_1* | DNA gyrase subunit A |
|  |  | *yciC_1* | Putative metal chaperone YciC |
|  |  | *treC* | Trehalose-6-phosphate hydrolase |
| 14 | 4303 | *malT_1* | HTH-type transcriptional regulator MalT |
| 15 | 5263 | *srmB_1* | ATP-dependent RNA helicase SrmB |
|  |  | *cdhR_2* | HTH-type transcriptional regulator CdhR |
| 16 | 4866 | *algC* | Phosphomannomutase/phosphoglucomutase |
|  |  | *mshD_5* | Mycothiol acetyltransferase |
|  |  | *lutP_2* | L-lactate permease |
| 17 | 8964 | *kshA* | putative 3-ketosteroid-9-alpha-monooxygenase, oxygenase component |
| 18 | 6025 | *ideR* | Iron-dependent repressor IdeR |
|  |  | *pdxH_2* | Pyridoxine/pyridoxamine 5'-phosphate oxidase |
| 19 | 7286 | *hpr* | Hydroxypyruvate reductase |
| 20 | 4275 | *trmL* | tRNA (cytidine(34)-2'-O)-methyltransferase |
|  |  | *betI_8* | HTH-type transcriptional regulator BetI |
| 21 | 6946 | *ydhP_2* | Inner membrane transport protein YdhP |
| 22 | 13608 | *panC* | Pantothenate synthetase |
|  |  | *nadB* | L-aspartate oxidase |
|  |  | *coaX* | Type III pantothenate kinase |
|  |  | *argA* | Amino-acid acetyltransferase |
|  |  | *lsr2_2* | Nucleoid-associated protein Lsr2 |
|  |  | *clpC1* | ATP-dependent Clp protease ATP-binding subunit ClpC1 |
| 23 | 4447 | *rbbA* | Ribosome-associated ATPase |
| 25 | 20903 | *ppgK* | Polyphosphate glucokinase |
|  |  | *ychF* | Ribosome-binding ATPase YchF |
|  |  | *hsdS* | Type-1 restriction enzyme EcoKI specificity protein |
|  |  | *xerC_2* | Tyrosine recombinase XerC |
|  |  | *rppH_7* | RNA pyrophosphohydrolase |
|  |  | *gpm2_2* | Acid phosphatase |
|  |  | *speE_3* | Polyamine aminopropyltransferase |
| 26 | 5515 | *iscS_2* | Cysteine desulfurase IscS |
|  |  | *sodF1* | Superoxide dismutase [Fe-Zn] 1 |
|  |  | *gabP_1* | GABA permease |
|  |  | *gabP_2* | GABA permease |
| 26 | 17310 | *glnA_1* | Glutamine synthetase |
|  |  | *nagR_2* | HTH-type transcriptional repressor NagR |
|  |  | *betI_13* | HTH-type transcriptional regulator BetI |
| 28 | 6343 | *nagR_2* | HTH-type transcriptional repressor NagR |
| 29 | 5131 | *gabD1_2* | Succinate-semialdehyde dehydrogenase [NADP(+)] 1 |
|  |  | *mutB* | putative methylmalonyl-CoA mutase large subunit |
| 30 | 4371 | *spk1* | Serine/threonine-protein kinase PK-1 |
|  |  | *thiG* | Thiazole synthase |
|  |  | *thiS* | Sulfur carrier protein ThiS |
|  |  | *hcnC* | Hydrogen cyanide synthase subunit HcnC |
| 31 | 5539 | *mcl2* | (3S)-malyl-CoA thioesterase |
|  |  | *smtB_2* | Succinyl-CoA--L-malate CoA-transferase beta subunit |
|  |  | *rbsK_2* | Ribokinase |
|  |  | *tri1_4* | ADP-ribosylarginine hydrolase Tri1 |
| 32 | 12585 | *ddc_2* | L-2,4-diaminobutyrate decarboxylase |
|  |  | *iucD_2* | L-lysine N6-monooxygenase |
|  |  | *cpdB* | 2',3'-cyclic-nucleotide 2'-phosphodiesterase/3'-nucleotidase |
|  |  | *pyk_2* | Pyruvate kinase |
|  |  | *pdtaR* | putative transcriptional regulatory protein pdtaR |
|  |  | *livF_1* | High-affinity branched-chain amino acid transport ATP-binding protein LivF |
|  |  | *lptB* | Lipopolysaccharide export system ATP-binding protein LptB |
| 33 | 10088 | *livH* | High-affinity branched-chain amino acid transport system permease protein LivH |
|  |  | *cas9* | CRISPR-associated endonuclease Cas9 |
|  |  | *spo0M* | Sporulation-control protein spo0M |
| 34 | 4415 | *lldR_2* | Putative L-lactate dehydrogenase operon regulatory protein |
|  |  | *ipuC* | Glutamate--isopropylamine ligase |
|  |  | *feaB* | Phenylacetaldehyde dehydrogenase |
|  |  | *lvr_2* | Levodione reductase |
| 35 | 9177 | *fadB2_1* | 3-hydroxybutyryl-CoA dehydrogenase |
|  |  | *xerC_3* | Tyrosine recombinase XerC |
| 36 | 4543 | *ribD* | Riboflavin biosynthesis protein RibD |
|  |  | *ribE* | Riboflavin synthase |
|  |  | *ribM* | Riboflavin/roseoflavin transporter RibM |
|  |  | *ribBA* | Riboflavin biosynthesis protein RibBA |
|  |  | *ribH* | 6,7-dimethyl-8-ribityllumazine synthase |
|  |  | *hisE* | Phosphoribosyl-ATP pyrophosphatase |
| 37 | 6048 | *btuD_21* | Vitamin B12 import ATP-binding protein BtuD |
|  |  | *pgm* | Phosphoglucomutase |
|  |  | *udgB* | Type-5 uracil-DNA glycosylase |
| 38 | 4221 | *tauD* | Alpha-ketoglutarate-dependent taurine dioxygenase |
|  |  | *nasC* | Assimilatory nitrate reductase catalytic subunit |
|  |  | *nasD_1* | Nitrite reductase [NAD(P)H] |
|  |  | *nasD_2* | Nitrite reductase [NAD(P)H] |
| 39 | 9413 | *spsB* | Signal peptidase IB |
|  |  | *ephD_2* | putative oxidoreductase EphD |
|  |  | *hmp_2* | Flavohemoprotein |
| 40 | 4648 | *fadA_2* | 3-ketoacyl-CoA thiolase |
| 41 | 5966 | *pikAV_2* | Thioesterase PikA5 |
|  |  | *qacA_6* | Antiseptic resistance protein |
|  |  | *tetC* | Transposon Tn10 TetC protein |
|  |  | *betI_16* | HTH-type transcriptional regulator BetI |
| 42 | 13883 | *cimA_2* | (R)-citramalate synthase |
|  |  | *fadA_11* | 3-ketoacyl-CoA thiolase |

**Table S3:** Genes potentially involved in stress response and marine adaptation in *Streptomyces albidoflavus* VIP-1.

| **Genes** | **Product** | **Potential role** |
| --- | --- | --- |
| *katA, katE* | Catalases | Protecting against oxidative stress. |
| *Ahp* | Alkyl hydroperoxide reductase |  |
| *sodN, sodF1* | Superoxide Dismutases |  |
| *nuoK, nuoH, nuoA, nuoB, nuoC, nuoD, nuoF, nuoG, , nuoL, nuoM, nuoN , nuoJ,* | NADH-quinone oxidoreductase subunits | Osmatic pressure resistance |
| *betP* | Glycine betaine transporter | Osmoprotectant transportation |
| *OpuCD, OsmX, OpuBB, OpuCA* | Osmoprotectant transportation system |  |
| *glnQ, glnH, glnM* | Glutamine ABC transportation systems |  |
| *oppA, oppC, oppD, oppF* | Oligopeptide transporter | Transportation of nutrients in the cell |
| *tap* | Multidrug efflux pump | Excretion of toxin and harmful substances |
| *cspA* | Cold Shock Protein | Stabilize mRNA and ribosomes at low temperatures, promoting protein synthesis. |
| *dps* | DNA-binding protein from starved cells | Protects DNA from oxidative damage. |
| *dnaK, dnaJ, clpB, clpX, clpP* | Molecular chaperones | Assist in protein folding and degradation, maintaining protein homeostasis under stress conditions. |
| *rnhA, recA, lexA* | DNA repair proteins | Repair DNA damage caused by various stress factors. |
| *yaaA. yfkM, yceC, ydaD, srkA, nhaX, yceD* | General stress proteins | Involved in various stress response pathways |

**Figure S1:** Genomic islands (GIs) identified using the IslandViewer 4 server in the genome of *Streptomyces albidoflavus* VIP-1. Wavy lines indicate contig boundaries.

**
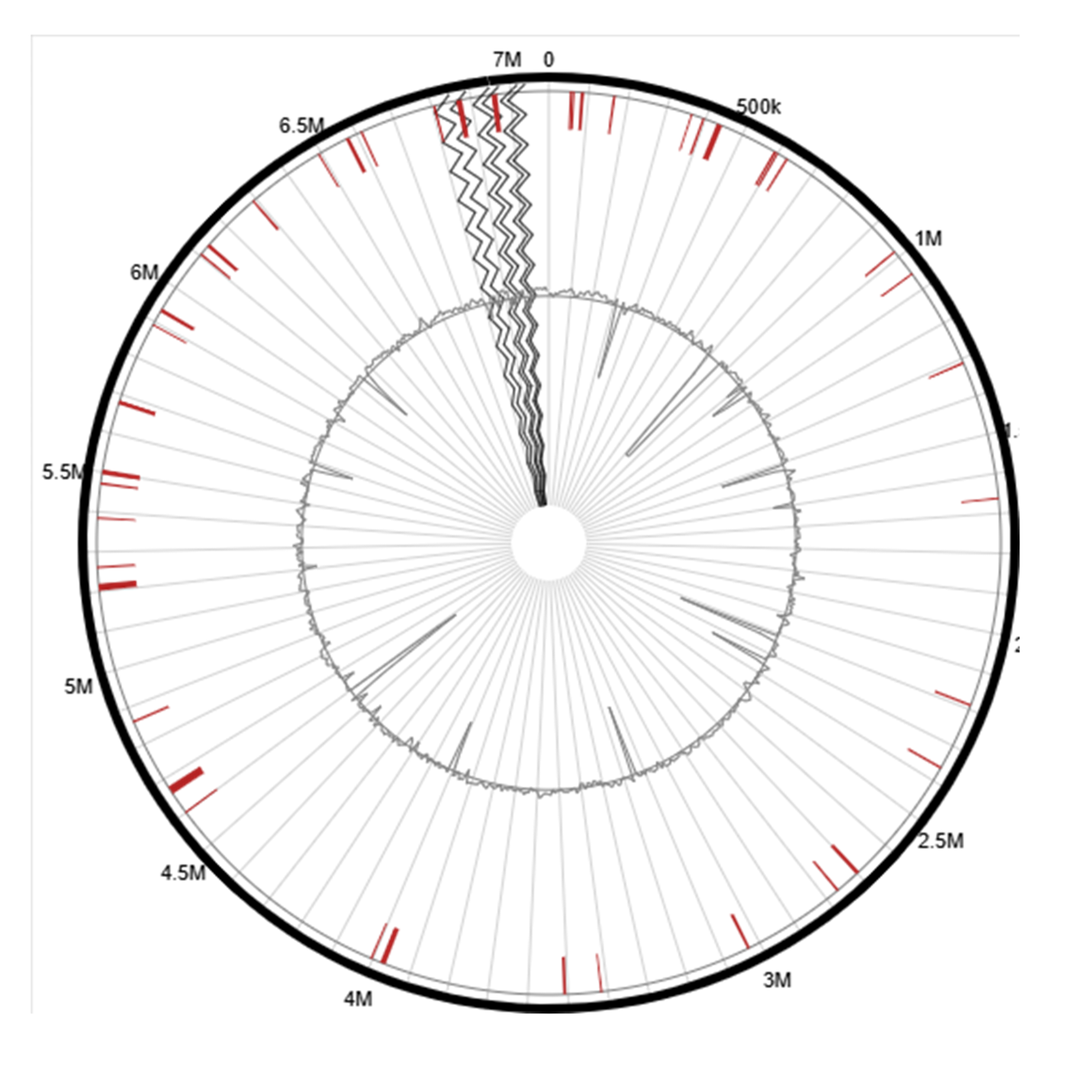
**

**Figure S2: (a)** Principal Component Analysis (PCA) of *Streptomyces albidoflavus* VIP-1 and five other *S. albidoflavus* strains based on the occurrence of selected genes potentially involved in marine adaptation. PC1 and PC2 explain 52.1% and 29.7% of the total variance, respectively. The ellipses represent a 95% confidence interval for cluster membership. **(b)** Heatmap showing the occurrence of genes potentially involved in marine adaptation across *S. albidoflavus* VIP-1 and five related strains. Rows were clustered using Euclidean distance and average linkage; columns were clustered using correlation distance and average linkage. Genes with uniform occurrence across all strains were excluded from the analysis.


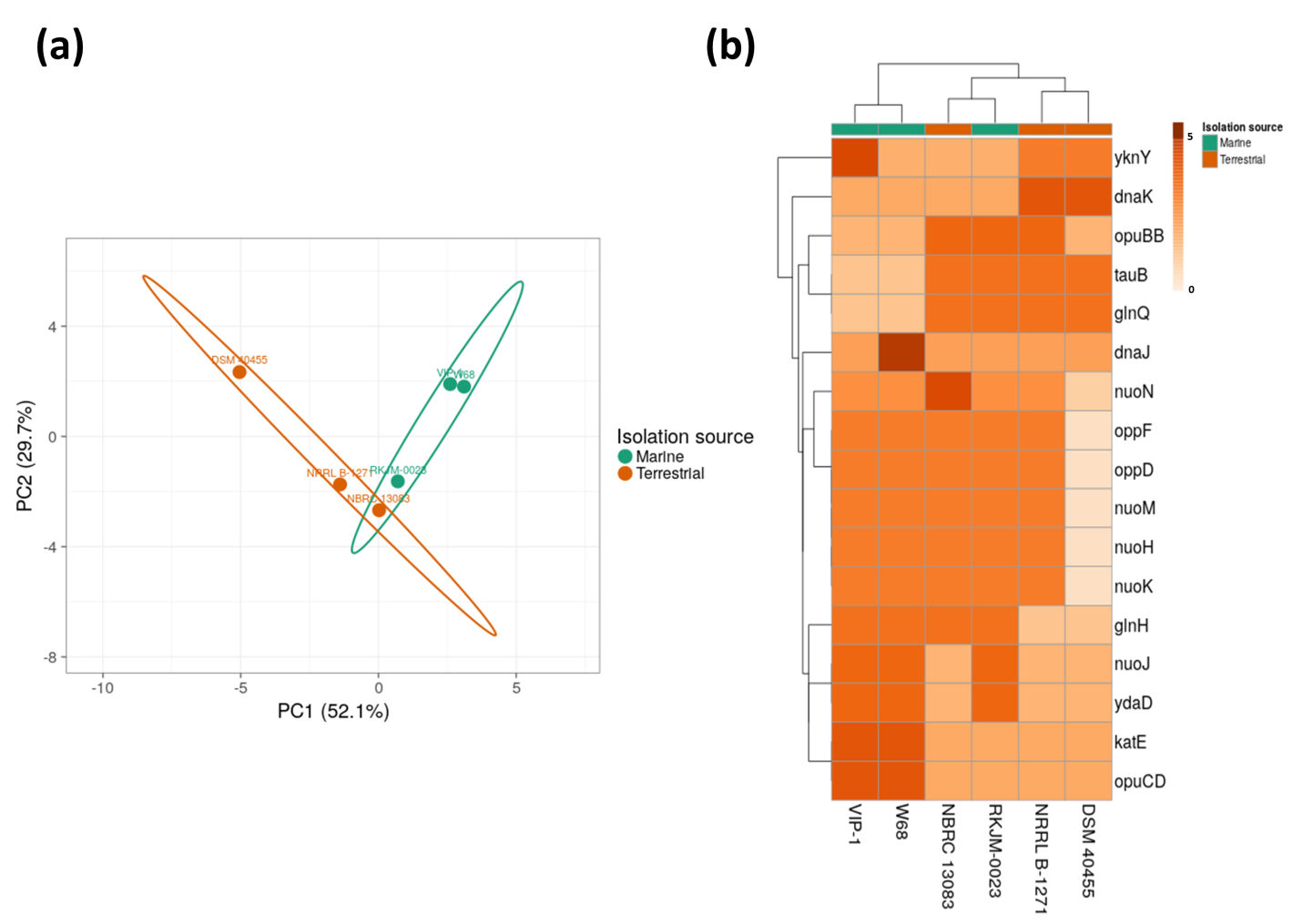

Supplement: Supplementary file 1 — Supplementary Material 1. [file 12866_2025_4109_MOESM1_ESM.docx]
